# Genome-wide identification of small GTPase gene family members in *Lentinula edodes* and the roles of *LeRho1* in biotic and abiotic stress responses

**DOI:** 10.1128/aem.01967-25

**Published:** 2026-03-18

**Authors:** Jiaxin Song, Tianwen Zhu, Ruiping Xu, Hairong Yin, Haiying Zhong, Jiyan Zhang, Yinbing Bian, Yuhua Gong, Lianfu Chen, Yan Zhou

**Affiliations:** 1 Plant Science and Technology College, Huazhong Agricultural University 467852, Wuhan, Hubei Province, China; 2 Hubei Hongshan Laboratory, Huazhong Agricultural University 47895, Wuhan, Hubei Province, China; 3 State Key Laboratory of Agricultural Microbiology, Huazhong Agricultural University 124443 , Wuhan, Hubei Province, China; Anses, Maisons-Alfort Laboratory for Food Safety, Maisons-Alfort, France

**Keywords:** *Lentinula edodes*, stress response, gene expression, small GTPase, overexpression

## Abstract

*Lentinula edodes* (*L. edodes*) is the largest proportion of edible mushrooms in China. However, the yield is highly susceptible to environmental stress. Factors such as high-temperature stress, low-temperature stress, *Trichoderma* infection, heavy metal stress, and light exposure all impact the growth status of *L. edodes* mycelium to varying degrees. Small GTPase is a small molecular switch involved in multiple biological processes of eukaryotes. The main functions of small GTPase in fungi include morphogenesis, secondary metabolism, vesicle trafficking, stress response, and virulence. However, the understanding of small GTPase in *L. edodes* is minimal. In this study, a total of 34 small GTPase genes in *L. edodes* were identified and clustered into five subfamilies, namely, *Rho*, *Ras*, *Arf*, *Rab*, and *Ran*. The 34 identified genes were phylogenetically analyzed and compared with those of various ascomycetes and basidiomycetes using the genome assembly and annotation databases of *L. edodes*. The results of expression patterns of 34 small GTPase genes under different biotic and abiotic stresses showed that most of these genes exhibited different degrees of responses to different stresses. The gene function analysis showed that the heat tolerance, resistance to *Trichoderma atroviride* (*T. atroviride*) infection, and light sensitivity of the *LeRho1* overexpression transformants were significantly higher than those of the control transformants. This study verified that *LeRho1* is an important stress-resistant gene in *L. edodes* and that this gene is distributed in edible fungi. This study verified that *LeRho1* is an important stress-resistant gene in *L. edodes* and that this gene is distributed in edible fungi. Our findings provide a theoretical basis for further research on the stress response mechanism of the small GTPases in large edible fungi.

**IMPORTANCE:**

This study verified that *LeRho1* is an important stress-resistant gene in *L. edodes* and that this gene is distributed in edible fungi. Clarifying the function of LeRho1 protein in the heat stress response of *L. edodes,* and analyzing the differentiation of the structure and function of small GTPase in fungi such as *L. edodes*, *Saccharomyces cerevisiae*, and *Aspergillus fumigatus*, is of great significance for elucidating the heat stress response mechanism of filamentous fungi of Agariales under heat stress and for conducting germplasm innovation for heat-tolerant edible fungi.

## INTRODUCTION

*Lentinula edodes*, a cornerstone species in the global edible fungi industry, valorizes agricultural waste through lignin degradation using extracellular enzymes (1, 2). Its cultivation faces five major environmental constraints: heat stress (30–42°C sensitivity), *Trichoderma* infection, cold (<5°C), photoinhibition, and heavy metals (1, 3). While fruiting requires 5–15°C diurnal fluctuations and light exposure, these same factors inhibit vegetative mycelial growth during the dominant (85%) 12- to 15-week cultivation phase (4, 5). Heat stress induces ROS-mediated cellular damage, disrupting membrane integrity, metabolic pathways (TCA cycle, sphingolipid synthesis), and carbon utilization (6, 7). Although HSPs stabilize IAA biosynthesis under thermal stress (8, 9), molecular mechanisms remain poorly characterized. *Trichoderma* co-infection under heat stress exacerbates economic losses through mycelial deformation and suppressed growth (10, 11). Current fungicide-dependent control (carbendazim/thiabendazole) raises biosafety concerns, with only *LeTLP1* identified as a resistance gene (10). Paradoxically, light and cold—essential for fruiting body maturation—suppress mycelial vitality during vegetative growth (5). Heavy metal tolerance mechanisms, including *LeAmy*-mediated cadmium resistance, remain secondary to improving mycelial resilience during the extended cultivation phase (3, 12). Developing stress-resistant mycelial strains represents a critical research frontier for sustainable shiitake production.

Small GTPases, evolutionarily conserved molecular switches governing cellular adaptation to environmental stress, have emerged as pivotal regulators across eukaryotes (13). Recent phylogenomic analyses reveal that fungal genome evolution involved punctuated gene family expansions alongside gradual loss of protist-derived genes, with extracellular proteins and nutrient coordination systems showing particularly rapid duplication rates (14). This genomic remodeling created specialized toolkits for sessile osmotrophic lifestyles, wherein small GTPase families likely played crucial roles in adapting to heterogeneous environments. The small GTPase superfamily comprises five subfamilies (Rho, Arf, Ras, Rab, and Ran) that coordinate essential processes through GTP/GDP cycling (15). Their evolutionary trajectory mirrors broader genomic patterns in fungi: while basal lineages retain ancestral opisthokont GTPase repertoires, derived species exhibit subfamily-specific expansions (14). Particularly in fungi, Rho-type GTPases demonstrate unique functional specialization shaped by these duplication events. Rho1 coordinates cell wall biosynthesis through β-glucan synthase activation while modulating MAPK-mediated stress signaling cascades (13, 16), exemplifying how gene family neofunctionalization underpins ecological adaptation.

In this study, we provide a comprehensive analysis of the small GTPase superfamily in *L. edodes*, encompassing phylogenetic relationships, expression profiling under multiple environmental stresses, and functional validation through genetic manipulation. We systematically identified 34 small GTPase family members and performed phylogenetic analysis to elucidate their evolutionary relationships with homologs in Ascomycetes and Basidiomycetes. This revealed distinct clades and conserved domains, highlighting functional diversification across fungal lineages. We then designed a robust expression profiling experiment to investigate the transcriptional responses of these genes to the five most prevalent environmental stresses in mushroom production: heat stress, cold stress, light inhibition, heavy metal stress, and *Trichoderma* infection. Our innovative sampling strategy captured dynamic expression patterns, revealing stress-specific and shared regulatory mechanisms. To functionally validate these findings, we developed a highly efficient overexpression vector for *L. edodes* and introduced the *LeRho1* gene into the YS3334 strain. Overexpression of *LeRho1* significantly enhanced thermotolerance, *Trichoderma* resistance, and light sensitivity, providing direct evidence of its role in stress adaptation. By integrating phylogenetic, RT-qPCR, and functional approaches, our study advances the understanding of fungal stress adaptation mechanisms and offers valuable targets for molecular breeding programs aimed at improving the sustainability and productivity of *L. edodes* cultivation.

## MATERIALS AND METHODS

### *L. edodes* and *Trichoderma* strains

*L. edodes* strain YS3334 was collected and identified by the Institute of Applied Mycology, Huazhong Agriculture University (17). The *T. atroviride* strain 92-1 was isolated and identified from logs with green mold disease in Suizhou, Hubei (18).

### Identification and functional annotation of *L. edodes* small GTPase gene family

*L. edodes* small GTPase gene family members were identified by referring to multiple databases. We downloaded all the required sequences and annotation files of *L. edodes* (W1-26) from MycoCosm of JGI (https://mycocosm.jgi.doe.gov/Lentinedodes1/Lentinedodes1.home.html) and NCBI (https://www.ncbi.nlm.nih.gov/), as well as seed files of the conserved domain (PF00071, PF0002, PF04670, and PF08477) of *L. edodes* small GTPase from Pfam (http://pfam.xfam.org/). HMMER (Hidden Markov Model, HMM) software was used to detect the conserved Pfam domain with default parameters (E-value <0.05) (19). Subsequently, conserved domains of sequences were aligned using local ClustalW (20). Based on the resultant ClustalW files, secondary retrieval was performed using hmm-search in HMMER software to obtain the *L. edodes* small GTPase gene as comprehensively as possible. Then, we verified the existence of the obtained *L. edodes* small GTPase conserved domains using the NCBI Batch CD-Search tool (Batch CD-Search: https://www.ncbi.nlm.nih.gov/cdd/), based on CDD v3.18 database (Table S1). The annotation information for *L. edodes* was extracted from the GFF files, and the result was visualized using TBtools (21).

### Analysis of gene structure and conserved motif of *L. edodes* small GTPase proteins

The gene structure display server (GSDS 2.0) (http://gsds.cbi.pku.edu.cn/) was used to analyze the gene structure of the *L. edodes* small GTPases by aligning their cDNA sequences to the corresponding genomic DNA sequences (22). Conserved motif analysis of *L. edodes* small GTPase proteins was performed using online multiple expectation maximizations for motif elicitation (MEME) (http://meme.nbcr.net/meme/cgibin/meme.cgi) with default parameters, and the maximum number parameter of motifs was set as 10 (23).

### Chromosomal location and characterizations of *L. edodes* small GTPase genes

Information on the chromosomal location of the *L. edodes* small GTPase genes was obtained from the NCBI database (BioSample ID: SAMN14591202, https://www.ncbi.nlm.nih.gov/datasets/genome/GCA015476405.1/), and the TBtools software was used to align these genes to different chromosomes (21). The amino acid length (aa), molecular weight, and theoretical isoelectric point (pI) of these *L. edodes* GTPase genes were estimated using ExPASY (http://web.expasy.org/protparam/).

### Analysis of putative *cis*-acting element of small GTPase genes in *L. edodes*

The 800 bp sequences upstream of the transcription start site of 34 predicted *L. edodes* small GTPase genes were downloaded from the JGI database. These obtained sequences were used for analyzing putative *cis*-acting elements related to stress response (such as heat stress and low-temperature stress) based on the PlantCARE database (http://bioinformatics.psb.ugent.be/webtools/plantcare/html/)

### Sampling strategy for environmental stress responses of small GTPase genes in *L. edodes*

*L. edodes* strain YS3334 was cultured at 25°C in MYG medium (containing 1% malt extract, 0.1% peptone, 0.1% yeast extract, and 2% glucose). Fresh mycelium blocks were cultured in a 9 cm petri dish containing MYG medium. After an 8-day dark culture, they were divided into different treatment groups (groups A, B, C, D, E). Group A was treated with 8 mm diameter *L. edodes* mycelium block on MYG medium for 7 days and then treated at 38°C for 30 min. Group B was treated with 8 mm diameter *L. edodes* mycelium block on MYG medium for 7 days and then treated at 4°C for 60 min. Group C was treated with an 8 mm diameter *L. edodes* mycelium block on MYG medium for 7 days and then under 3,500 lx light for 60 min. In Group D, fresh 8 mm diameter *L. edodes* mycelium blocks were inoculated into MYG medium containing cadmium ions. For group E, an 8 mm diameter block of *L. edodes* mycelium was inoculated 10 mm away from the edge of a petri dish. The dish was then cultured at 25°C for 7 days. After that, an activated 8 mm diameter block of *Trichoderma* mycelium was inoculated 10 mm away from the edge of the petri dish at one end of the petri dish diameter (opposite to where the *L. edodes* mycelium block was inoculated). The petri dish was incubated at 25°C for 24 h in the dark to allow the *Trichoderma* mycelium to come into contact with the *L. edodes* mycelium (18). For each treatment group, *L. edodes* mycelium was cultivated on three petri dishes. After treatment, the mycelia from the dishes were combined to form a single sample for RNA extraction.

### RNA isolation and cDNA synthesis

Total RNA was extracted from 50 to 100 mg of *L. edodes* mycelium using RNAiso Plus reagent (Takara, Dalian, China), according to the manufacturer’s instructions. RNA concentration and purity were measured using the DS-11+ Spectrophotometer (DeNovix, USA), and the integrity was confirmed using 1.2% agarose gel. According to the protocol described in the manual, 1 μg of RNA from each sample was reverse-transcribed into cDNA using Highscript II Reverse Transcriptase (Vazyme, Nanjing, China), and the resulting cDNA was dissolved in 200 μL of water.

### qRT-PCR analysis

In this experiment, we analyzed the expression of 34 *L. edodes* small GTPase genes following different stress treatments using quantitative RT-PCR (qRT-PCR). The qRT-PCR was performed using CFX Connect Real-Time PCR system (BIO-RAD) in a 10 μL reaction system containing 5 μL of 2×AceQ qPCR SYBR Master Mix (Vazyme, Nanjing, China), 3 μL of nuclease-free water, 1 μL (5 ng/μL) cDNA, and 0.5 μL each of the forward and reverse primer (10 mM). The RT-qPCR conditions were as follows: predenaturation at 95°C for 3 min, 40 cycles of denaturation at 95°C for 20 s, followed by annealing at 60°C for 30 s, three technical repetitions per reaction. Four genes, *Actin* (JGI: LE01Gene01050), *28S* (JGI: LE01Gene02296), *EF* (JGI: LE01Gene03252), and *Pma* (GenBank ID: AF146054), were selected as candidate reference genes on the basis of previous studies (24, 25). RefFinder, a web-based tool integrating geNorm, NormFinder, BestKeeper, and the comparative ΔCt method, was used to evaluate the expression stability and reliability of candidate reference genes (26). According to the comprehensive ranking of different reference genes or gene combination, the optimal reference genes were selected to calculate the relative expression level of 34 small GTPase genes. The generation and analysis of gene expression data in this study are stored in the Gene Omnibus database [GEO: GSE219161]. Due to the large extremes in gene expression data, the data were normalized by treatment sample (column-wise normalization) to ensure that the gene expression values follow a normal distribution. The relative expression analysis was performed using the “scale()” function in R language (27). Then, the normalized data were used to create a heatmap.

### Phylogenetic analysis

The phylogenetic tree was constructed using a maximum likelihood estimation method with a bootstrap of 1,000 and a minimum correlation coefficient of 0.90 using IQ-TREE. Maximum likelihood (ML) analysis of 306 small GTPase protein sequences was performed using IQ-TREE v2.2.0 based on an AVX + FMA - 6 thread model determined by jModelTest version 2.1.4 (28, 29). The approximate likelihood ratio test (aLRT) of all the samples was conducted with 1,000 replicates. Phylogenetic trees were modified using the online tool (iTOL) (https://itol.embl.de) (30). In order to highlight the clustering effect of homologous proteins in different species, the branch length of the phylogenetic tree was not taken into consideration.

### Construction of *LeRho1* overexpression and fungal transformation

**Fig 1:**
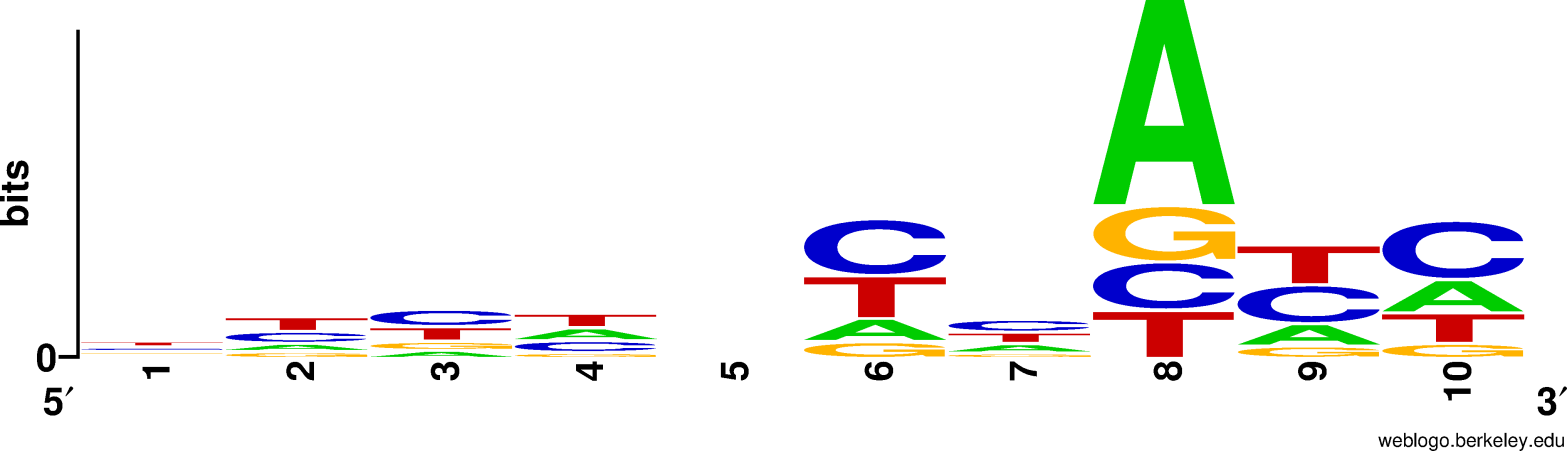
Upstream sequence of the start codon of *L. edodes*.

We have constructed vectors for the expression of both LeRho1-GFP fusion proteins and *LeRho1*-FLAG fusion proteins (Fig. 6A). These vectors facilitate the study of the *LeRho1* function and its role in the stress response of *L. edodes*. The *LeRho1* overexpression vector was constructed by following the published protocols described by Yan et al. (31). The CaMV 35S promoter of pCAMBIA1300 was replaced with the *LeEF1α* (*L. edodes* elongation factor 1 alpha) promoter in order to produce the pCAMBIA1300-E vector. The *LeEF1α* promoter sequence was PCR-amplified from the DNA of *L. edodes* strain W1-26 with the B-ef1a-F and AB-hyg-ef1a-R primers (Table S2). The full length of the *LeRho1* gene was amplified from *L. edodes* strain *YS3334* cDNA with the HR-Rho1-GFP-F, HR-Rho1-GFP-R, pOE-Rho1-Flag-F, and pOE-Rho1-Flag-R (Table S2), which contained the homologous arms, and then ligated into the pCAMBIA1300-E vector digested with *EcoR* I and *BamH* I in order to generate the overexpression vector pCAMBIA1300-E-Rho1-GFP and pCAMBIA1300-E-Rho1-FLAG. Furthermore, we concatenated the Kozak (-GCCATCATG-) sequence of *L. edodes* behind the promoter (Fig. 1). This Kozak sequence was composed by statistically combining the high-frequency bases of the six bases upstream of the translation initiation sites of the 200 genes with the highest expression levels in the published transcriptome of *L. edodes* (8). The statistics were compiled using WebLogo3 software (32). All constructs were assessed by sequencing analysis and transferred into *L. edodes* strain YS3334 through *A. tumefacien*s EHA105 infection. The fluorescence of green fluorescent proteins (GFP) was observed 2 to 3 days after mycelium inoculation through an FV3000 series (Confocal Laser Scanning Microscope, Olympus Corporation, Japan). Western blotting preparation steps were performed as described in reference 33).

### Establishment of a comprehensive stress resistance assessment protocol for *L. edodes*

#### Assessment of *L. edodes* heat/cold tolerance and light sensitivity

**Fig 2:**
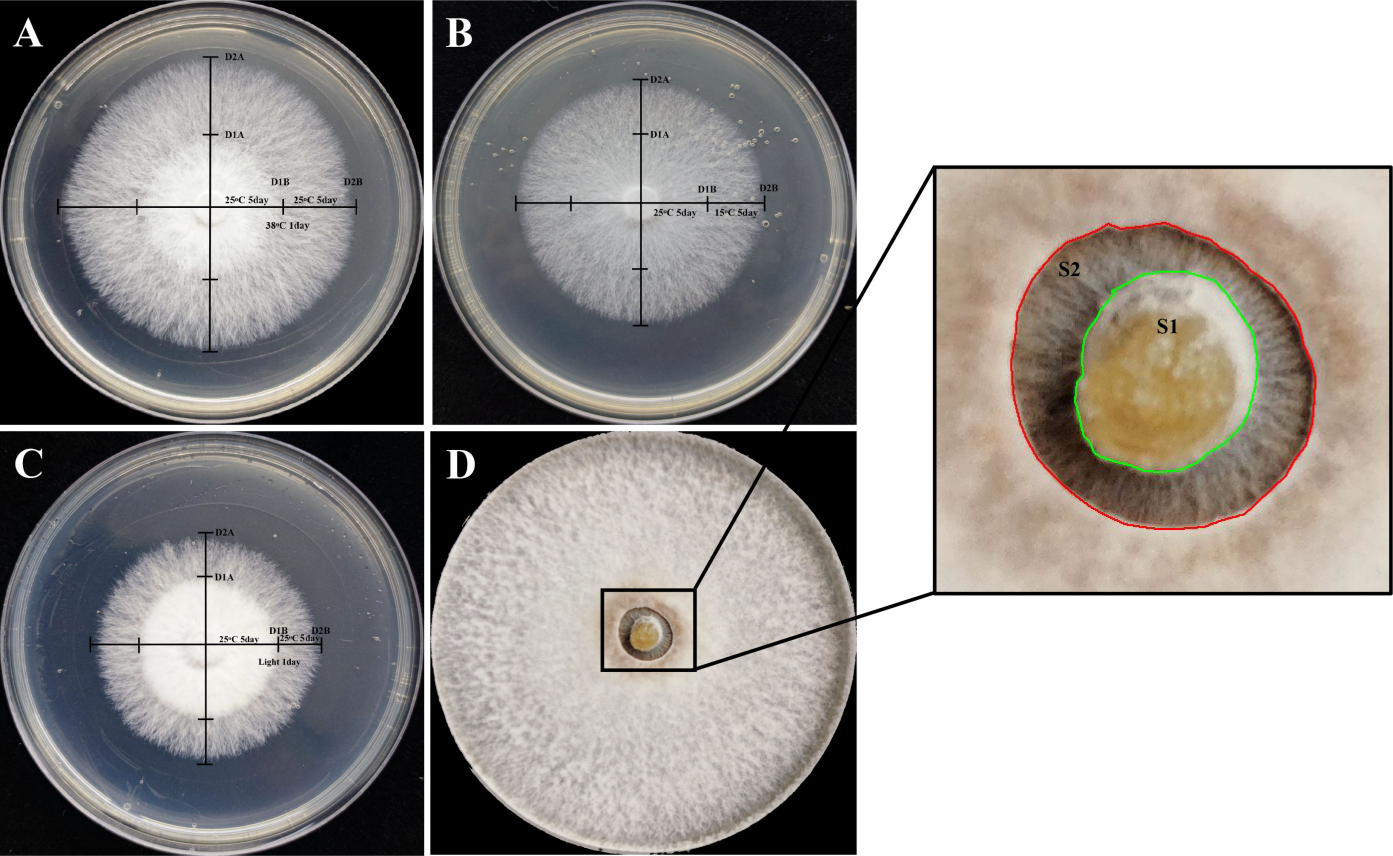
Schematic diagram for measuring the growth of *L. edodes* mycelium. (**A**) The diameter of the resumed growth of the *L. edodes* mycelium after one-day treatment at 38°C was measured using the cross-over method. (**B**) The growth diameter of *L. edodes* mycelium after 5 days of dark culture at 15°C was measured using the cross-over method. (**C**) The diameter of mycelial regrowth after one-day treatment under a light intensity of 3,500 lux was measured using the cross-over method. (**D**) The area of the *L. edodes* mycelium infected by *Trichoderma* after 5 days of infection was measured using the cut-and-patch method.

*L. edodes* mycelium blocks were inoculated into MYG agar plates and incubated in darkness at 25°C for 5 days to complete the pre-cultivation process. After pre-cultivation, the cross-over method was used to measure the two perpendicular diameters of the mycelium, recorded as D1A and D1B. Plates were subjected to the following treatments. (i) Heat tolerance test: incubation at 38°C for 1 day, followed by 5 days of recovery at 25°C (Fig. 2A). (ii) Cold tolerance test: incubation at 15°C for 5 days, followed by 5 days of recovery at 25°C (Fig. 2B). (iii) Light sensitivity test: exposure to light with an intensity of 3,500 lx at 25°C for 1 day, followed by 5 days of recovery in darkness at 25°C (Fig. 2C).

Following the recovery period, mycelial diameters were remeasured using the cross-over method, with each sample recording diameters D2A (primary axis) and D2B (orthogonal axis). Growth increments were calculated as DA = D2A − D1A and DB = D2B − D1B. Relative growth rate (RGR) was determined using the simplified formula RGR (%) = (ΔD / D₁) × 100, where for the horizontal axis ΔD = DA and D₁ = D1A, while for the vertical axis ΔD = DB and D₁ = D1B (note: formula simplification valid since both recovery days and pre-cultivation days equal 5 days). Statistical analysis incorporated five biological replicates and two directional measurements per replicate, yielding 10 data points per treatment group.

#### Evaluation of *Trichoderma* resistance in *L. edodes*

*L. edodes* was inoculated on MYG agar plates and incubated in darkness at 25°C for 10 days. Subsequently, the central portion of the aged 8 mm diameter *L. edodes* mycelium block was removed, and a similarly sized, activated *T. atroviride* mycelium block was inoculated in its place. After 5 days, photographs were taken to calculate the area (S) of the *L. edodes* mycelium infected by *T. atroviride* using ImageJ software. A smaller area (S) indicates a stronger resistance of the *L. edodes* to *T. atroviride* infection (Fig. 2D). The data on mycelium diameter and *Trichoderma* area are presented in Table S3. Images showing mycelium recovery growth are presented in Fig. S1 to S4.

#### Statistical analysis

After calculations, GraphPad Prism 10.3.0 software was used to perform one-way ANOVA to analyze the significance of differences among the groups. Significance level is set at *P* < 0.05. This experimental design evaluates the tolerance of *L. edodes* to different stress conditions by measuring mycelium growth recovery. Strict control of environmental factors, such as temperature and light, ensures the accuracy of the results.

## RESULTS

### Identification of 34 small GTPase family members in *L. edodes*

**Fig 3:**
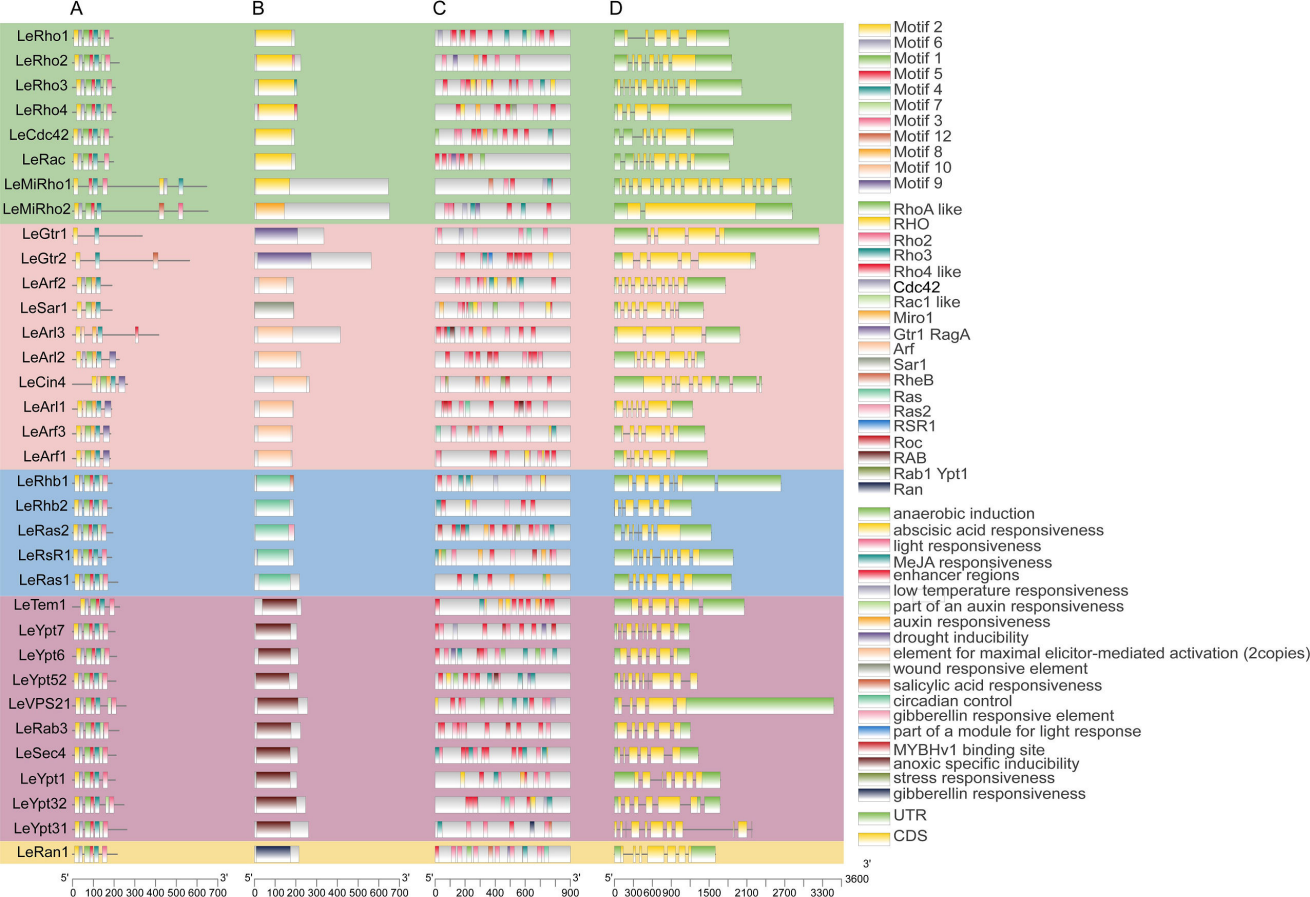
Distribution of conserved motifs, conserved domains within proteins, *cis*–acting elements of promoter sequences (800 bp), and gene structure of *L. edodes* small GTPase genes in *L. edodes*. (**A**) Schematic diagram of the conserved motifs in the *L. edodes* small GTPase proteins. Each motif is represented by a number in the colored box. The black lines represent the non-conserved sequence. A scale of gene and protein length is shown at the bottom. (**B**) Analysis of *L. edodes* small GTPase protein by NCBI’s conserved domain database. (**C**) The *cis*-acting elements of promoter sequences (800 bp) of 34 *L. edodes* small GTPase genes are analyzed by PlantCARE in *L. edodes*. (**D**) Gene structural analysis.

**Fig 4:**
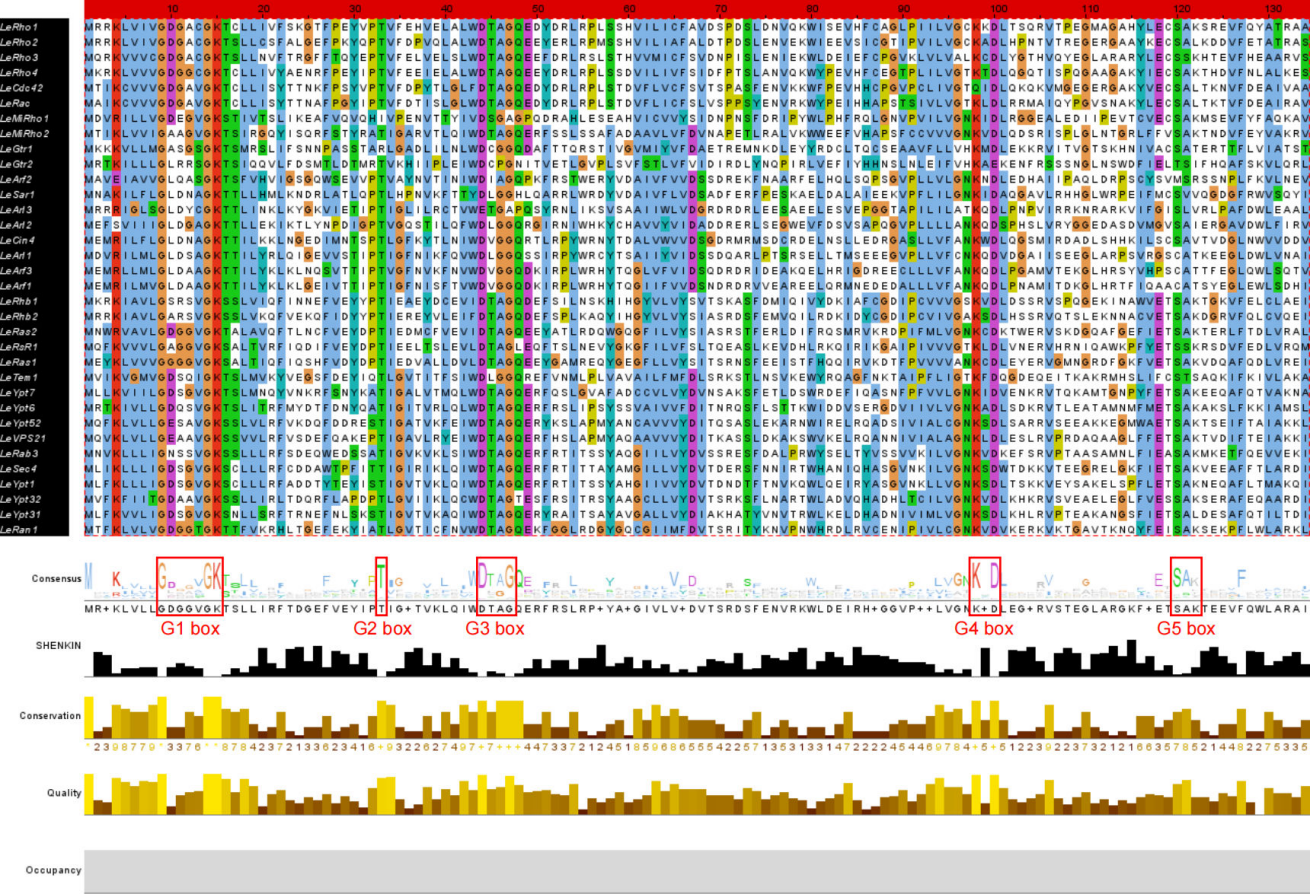
Analysis of conserved sequence of putative small GTPases in *L. edodes*. G1 box motif: GXXXXGK[T/S]; G2 box motif: T; G3 box motif: DxxG; G4 box motif: [N/T]KXD; G5 box: [C/S]A[K/L/T].

To more comprehensively identify the small GTPase genes, we performed a local HMM search based on the HMM files (PF00071, PF0025, PF04675, and PF08477) against the previously reported genome of *L. edodes* available in JGI MycoCosm database (*L. edodes* W1-26 v1.0) with default parameters. A total of 198 putative small GTPase proteins in *L. edodes* were predicted. Conserved domain analysis was performed for all predicted proteins using Batch CD-search analysis (https://www.ncbi.nlm.nih.gov/Structure/cdd/wrpsb.cgi), and the domain was visualized using TBtools (Fig. 3B). Then, based on conserved domain analysis of predicted proteins, redundant sequences were removed. Finally, 34 *L. edode*s small GTPase superfamily members were identified. All the *L. edodes* small GTPase superfamily members had G-box motifs (Fig. 4), and these G-box motifs were further visualized by DNAMAN v9.0.

The analysis results of *L. edodes* small GTPase protein characteristics indicated that the *L. edodes* small GTPase protein length ranged from 128 (for LeRan) to 609 (for LeMitRho2) amino acid residues. The molecular weight of the protein (MW) ranged from 1.4 kDa (for LeRan) to 6.6 kDa (for LeMitRho2) (Table 1). The isoelectric point (pI) of all 34 *L. edodes* small GTPase members varied from 4.75 (for LeYpt7) to 9.32 (for LeArf1), and that of 29 *L. edodes* small GTPase superfamily members was lower than 7. The *L. edodes* small GTPase family comprises 19 members with instability indices greater than 40. The aliphatic index of 27 *L. edodes* small GTPase proteins was greater than 80. The results of instability indices and aliphatic index suggest that these proteins have good stability, which is advantageous for their normal functioning under stress conditions. The average hydropathicity value of 34 *L. edodes* small GTPase proteins was below 0, indicating their high hydrophily.

**TABLE 1:** Characteristics of identified *L. edodes* small GTPase

Protein name	Number of amino acid	Molecular weight	Theoretical pI	Instability index	Aliphatic index	Hydropathicity
LeSec4	208	23,098.01	5.29	42.33	84.42	−0.327
LeYpt7	203	22,969.85	4.75	35.08	76.8	−0.38
LeYpt6	211	23,469.59	5.29	44.57	79.48	−0.298
LeCdc42	192	21,281.56	5.81	30.71	89.69	−0.075
LeYpt52	207	22,807.75	6.42	40.4	74.54	−0.454
LeRac	196	21,629.94	8.53	30.55	88.01	−0.196
LeArf6	181	20,658.75	5.95	35.7	101.27	−0.138
LeRho1	194	21,568.98	6.89	53.98	93.35	−0.125
LeArf1	182	20,629.87	9.32	43.47	87.8	−0.369
LeYpt31	211	22,981.81	5.95	27.87	88.25	−0.208
LeRab3	222	24,224.2	5.35	45.29	80.72	−0.287
LeRho4	182	20,553.56	7.67	42.98	86.76	−0.351
LeArl1	187	20,679.7	5.12	48.51	100.53	−0.053
LeYpt1	232	26,103.64	7.65	39.84	82.72	−0.237
LeArf2	182	20,552.48	7.01	35.87	90.44	−0.007
LeYpt32	246	27,137.47	6.6	43.49	81.3	−0.397
LeRas5	217	24,007	5	37.45	72.67	−0.389
LeVPS21	256	27,237.36	4.93	47.26	77.85	−0.397
LeRho3	229	25,942.51	6.39	39.36	90.13	−0.172
LeRhb1	189	20,663.64	5.48	33.46	92.28	−0.024
LeRho2	223	24,720.08	5.87	42.06	76.95	−0.406
LeCin4	169	19,019.52	5.56	33.67	91.12	−0.292
LeRsR1	187	21,192.55	8.97	40.7	102.62	−0.129
LeMiRho2	601	66,153.2	6.42	64.63	54.36	−0.859
LeSar1	189	21,507.84	5.66	46.38	106.3	−0.01
LeTEM1	225	24,917.6	6.43	36.09	88.36	−0.007
LeRas9	192	21,901.08	8.62	41.45	79.69	−0.393
LeRhb2	202	22,855.35	6.83	47.83	98.37	−0.077
LeArl2	414	46,218.74	8.19	56.3	84.78	−0.25
LeMiRho1	627	70,054.88	5.49	36.68	89.55	−0.183
LeGtr1	334	37,205.46	6.16	53.31	84.67	−0.113
LeRan	128	14,696.8	5.71	33.45	87.58	−0.393
LeArl3	155	17,853.88	5.58	54.33	80.45	−0.686
LeGtr2	562	61,438.07	6.32	59.08	84.96	−0.209

### Analysis of gene structure, chromosomal location, and conserved motif of *L. edodes* small GTPase superfamily members

The exon and intron structures of *L. edodes* small GTPase genes in *L. edodes* were visualized using TBtools software. As shown in Fig. 3E, the *L. edodes* small GTPase genes within the same subfamily had similar gene structures, and these *L. edodes* small GTPase genes were highly similar in exon number, exon pattern, and exon length. Most *L. edodes* small GTPase genes exhibited longer 3′UTRs than 5′UTRs, and *LeRho4*, *LeGtr1*, *LeRhb1*, and *LeVps21* had much longer 3′UTRs than other members. The gene structure of the Arf subfamily was quite different from that of four other subfamilies (Except for LeMitRho1\2), and the exons of *LeArf2, LeSar1*, and *LeArl1* members were much longer than those of other family members. *LeArf2* had 11 introns, far more than other family members. Notably, the protein length of *LeMitRho2* and *LeMiRho1* (a mitochondrial small GTPase protein) was much longer than that of others. There were 16 exons in *LeMitRho1*, whereas *LeMitRho2* contains two, making them the two most special small GTPase proteins.

The type and structure of protein motif determine its function. In order to determine the evolutionary relationship of these small GTPase proteins, this study identified the type of small GTPase protein motifs in *L. edodes* using the online MEME program and visualized their structure with TBtools tool. As shown in Fig. 3A, a total of 10 conserved motifs were identified, and these motifs exhibited basically identical structures. Almost all these 10 motifs were concentrated at the C-terminus of the amino acid sequence, indicating that the C-terminal of *L. edodes* small GTPase was the conserved GTP-binding region, and no motifs were present at N-terminal, suggesting that the N-terminal was the main functional differentiation region of *L. edodes* small GTPase.

**Fig 5:**
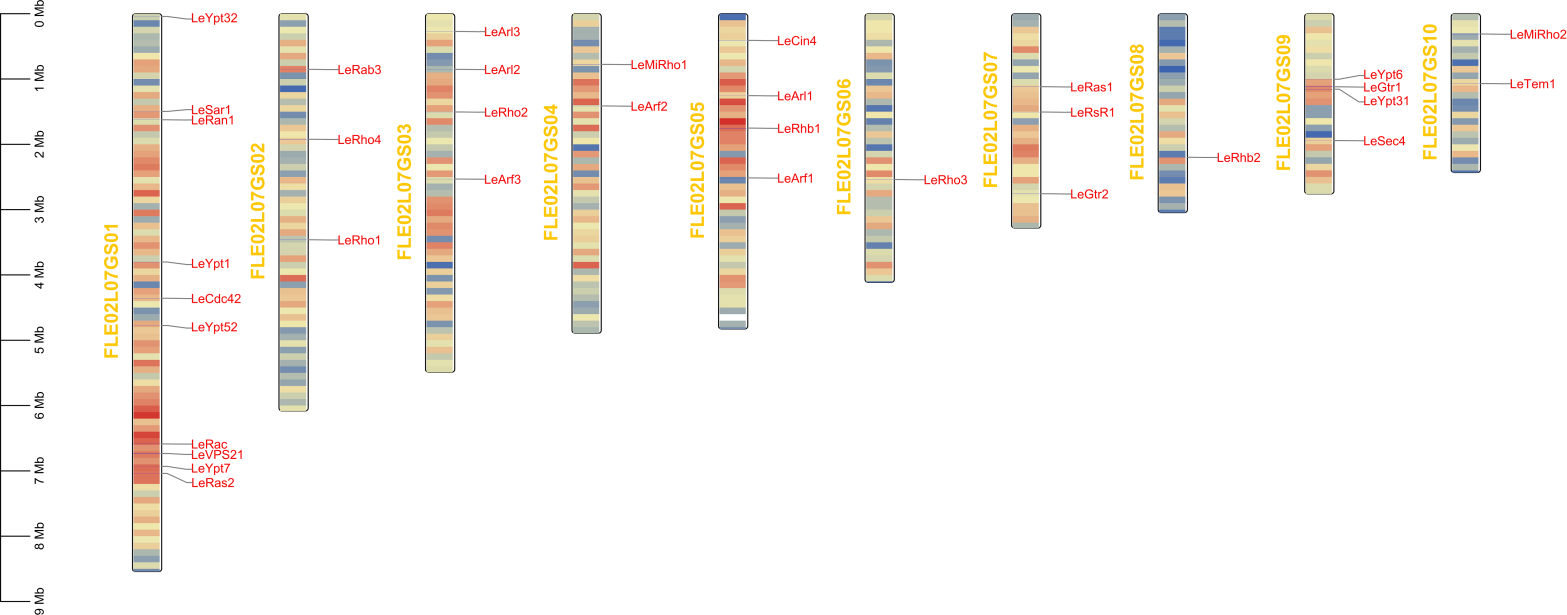
Chromosomal distribution and position of 34 *L. edodes* small GTPase genes identified in the *L. edodes* genome. The colors of the 10 chromosomes are rendered according to gene density. Yellow words represent the names of chromosomes in the *L. edodes* genome data.

The results of chromosome localization showed that 34 genes of *L. edodes* small GTPase genes were distributed evenly on 10 chromosomes and were located in the region with moderate gene density (Fig. 5). Among them, Chromosome 1 contained the largest number of *L. edodes* small GTPase genes (six genes). Chromosome 6 contains the least *L. edodes* small GTPase gene (only one gene) (Fig. 5). Chromosome localization results showed that there was no typical tandem repeat structure of small GTPase protein in *L. edodes*, indicating that the expansion of small GTPase protein family in *L. edodes* might have resulted from whole-genome duplication event during species evolution.

### *L. edodes* small GTPase family members are clustered into five subfamilies by phylogenetic analyses

**Fig 6:**
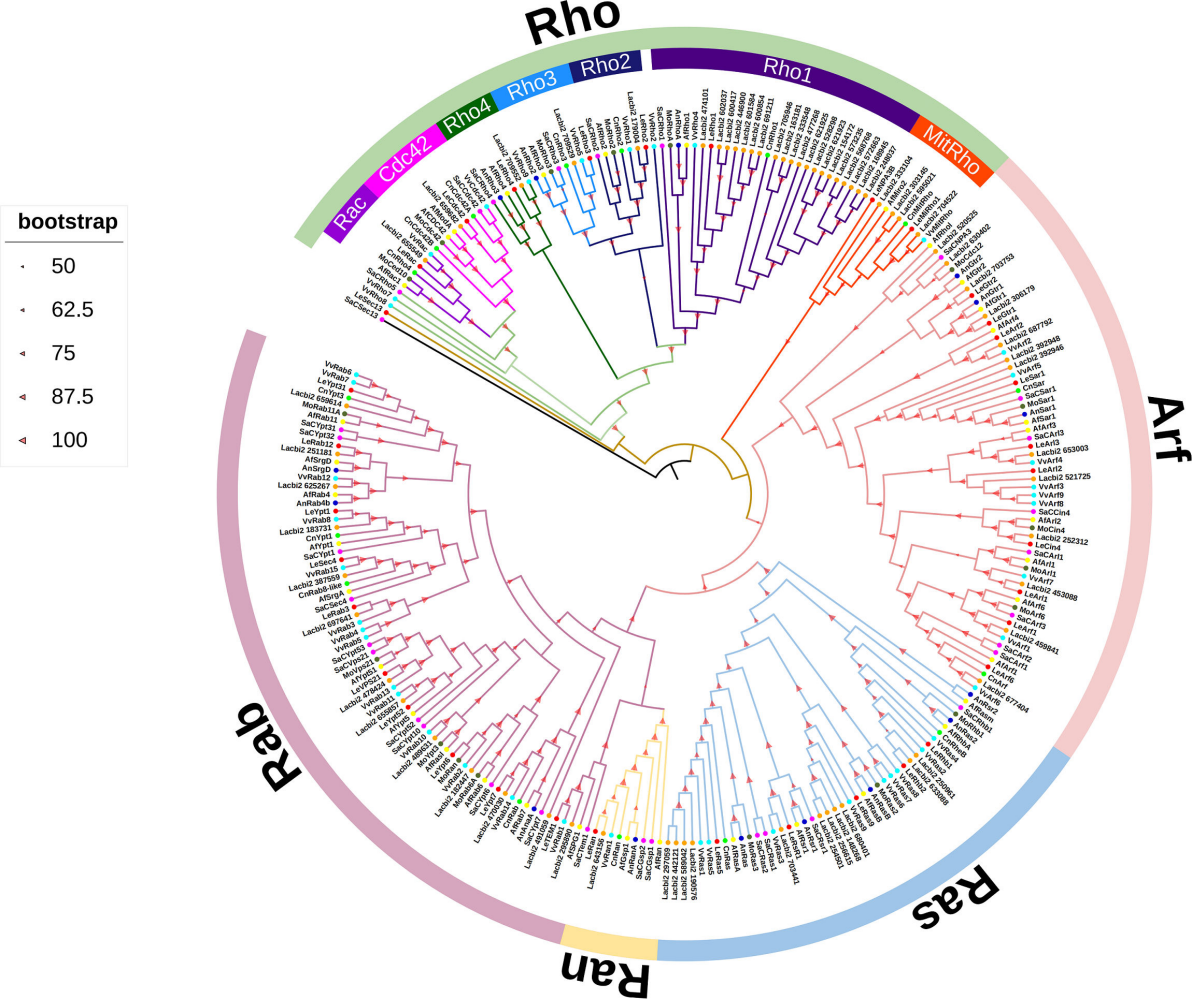
Phylogenetic tree and subfamily classifications of small GTPase proteins in *L. edodes*, *V. volvacea*, *L. bicolor*, *A. fumigatus*, *S. cerevisiae*, *A. nidulans*, *M. oryzae,* and *C. neoformans*. Phylogenetic tree constructed using small GTPase proteins from *L. edodes* (red circle), *V. volvacea* (cyan circle), *L. bicolor* (orange circle), *A. fumigatus* (yellow circle), *S. cerevisiae* (purple circle), *A. nidulans* (blue circle), *M. oryzae* (deep green), and *C. neoformans* (green circle). The phylogenetic tree was constructed using IQ-TREE v 2.2.2 and using the maximum likelihood method. Each subfamily member uses different color labels. All branch bootstrap values are greater than 50, using red triangles of different sizes.

To reveal the evolutionary relationship of *L. edodes* small GTPase proteins, the amino acid sequences of small GTPase proteins from three basidiomycetes (*Volvariella volvacea*, *Laccaria bicolor*, and *Cryptococcus neoformans*) and four ascomycetes (*Aspergillus fumigatus*, *Saccharomyces cerevisiae*, *Aspergillus nidulans*, and *Magnaporthe oryzae*) were selected to construct the phylogenetic tree. The *S. cerevisiae Sec13* and *L. edodes LeSec13* were used as outgroups. The phylogenetic tree showed that the small GTPase protein superfamily of *L. edodes* clustered into five clades, and these five clades were named Rho, Arf, Ras, Rab, and Ran according to the evolution time of branch nodes with Ran clade tightly close to Rab clade (Fig. 6). There were 7 members for the Rho clade, 10 for Arf, 5 for Ras, 10 for Rab, and only 1 for Ran (Fig. 6). Since all the members of the *S. cerevisiae* small GTPase protein superfamily have been systematically studied, small GTPase protein superfamily members of *L. edodes* were named after their orthologous genes in *S. cerevisiae* (16).

Small GTPase proteins of all species (three basidiomycetes and four ascomycetes) were also clustered into five clades. There were fewer small GTPase family members in ascomycetes than in basidiomycetes, and the number of small GTPase family members was proportional to their respective genome size, indicating the expansion of the genomes of these species, the increase in their signal network complexity, and the enhancement of environmental adaptability. All subfamilies were clustered into a branch, and the homologous genes of different species were clustered together (Fig. 6). There were fewer small GTPase family members in ascomycetes than in basidiomycetes, and the number of small GTPase family members was proportional to their respective genome size, indicating the expansion of the genomes of these species, the increase in their signal network complexity, and the enhancement of environmental adaptability. Among five subfamilies, the Rho subfamily exhibited the best clustering uniformity, and the small GTPase proteins with closer genetic relationships were clustered together. Rho2 and Rho3 displayed the most similar structure. The small GTPase proteins of *L. edodes* were close to those of two basidiomycetes (*V. volvacea*, *L. bicolor*) and *C. neoformans*, but distant from those of four ascomycetes (Fig. 6).

In recent years, a novel class of large Rho small GTPase called “MitRho” has been identified. The phylogenetic tree reveals that MitRho clusters with other Rho subfamilies, forming two distinct branches (Fig. 6). Among basidiomycetes, MitRho has been identified in fungi, such as *L. edodes*, *V. volvacea*, and *L. bicolor*. Specifically, *L. bicolor* possesses up to four *MitRho*, while *L. edodes* has two, *V. volvacea* has only one, and *C. neoformans* has only one. In ascomycetes, only *A. fumigatus* has a single *MitRho*. Notably, *MitRho* is absent in *S. cerevisiae*, *M. oryzae*, and *A. nidulans*. All fungi species examined in this study contain both Rho1 and CDC42, which are the most conserved and extensively studied small GTPase. These proteins play crucial roles in various cellular processes, and their disruption often leads to lethality in many fungi. Interestingly, other small GTPase family members show varying degrees of presence or absence across different species. Notably, the number of small GTPases in *V. volvacea* (43) and *L. bicolor* (67) far exceeds that in other species, correlating with the complexity of their life histories. Remarkably, *L. bicolor* has an astonishing 19 homologs of Rho1. The evolutionary tree further reveals that Rho proteins form the primary branch, with MitRho and Arf proteins in the secondary branch, Ras and Rab proteins in the tertiary branch, and Ran proteins positioned below Rab in the quaternary branch. Overall, this tree provides insights into the evolutionary relationships among subfamilies of fungal small GTPase.

### Response elements are identified from *L. edodes* small GTPase gene family promoter

To explore the dynamics of small GTPase gene expression in response to environmental stress, the *cis*-acting elements in the promoter region of small GTPase genes were identified and analyzed. As a result, three types of putative *cis*-elements were detected in the 800 bp gene upstream (Fig. 3C). The main type was stress-responsive elements, including light-responsive element, drought-inducible element, low-temperature responsive element, anoxic specific inducible element, wound-responsive element, and MYB element. The second type consisted of hormone-responsive elements, including auxin-responsive elements, MeJA-responsive elements (methyl jasmonate response), gibberellin-responsive elements, and TCA-responsive elements (salicylic acid response). The third type was the enhancer regions. All the promoter regions of small GTPases in *L. edodes* are rich in enhancer regions. Among them, within the promoter range of *LeRho1*, stress responsiveness, low temperature responsiveness, light responsiveness, and circadian control were identified. It is worth noting that six enhancer regions were also identified in its promoter, which is in accordance with the data indicating an extremely high expression level of this gene in the published transcriptome (8).

### Expression profiles of *L. edodes* small GTPase genes under different stress

**Fig 7:**
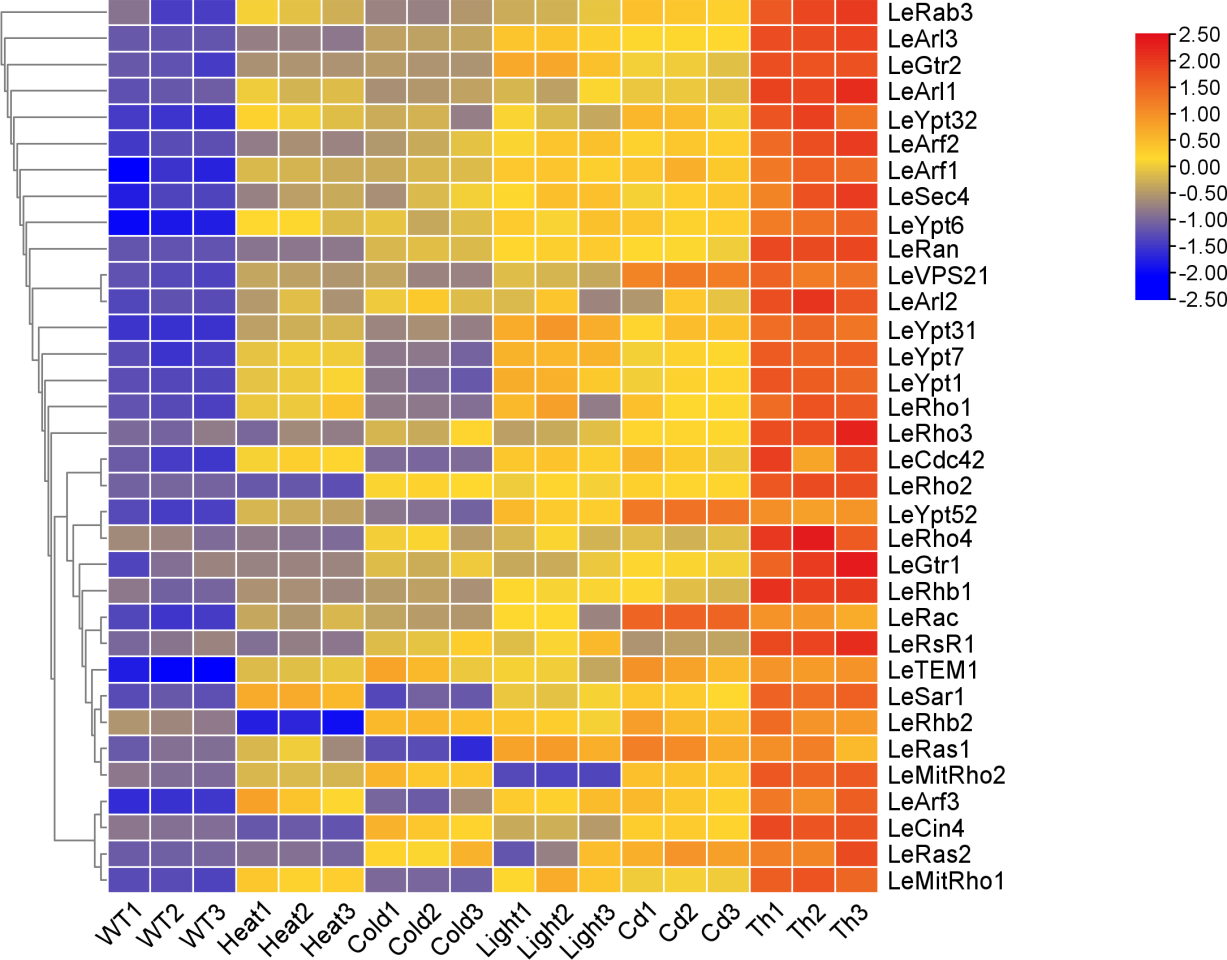
Expression analysis of the *L. edodes* small GTPase genes in different treatments. Expression analysis of the *L. edodes* small GTPase in different stress treatments using qRT–PCR. The displayed values represent the relative gene expression levels of stress-treated samples compared to control samples, which were subsequently standardized using Min-max normalization.

Some members of the small GTPase protein family have been reported to participate in the signal transduction of fungal responses to external environmental stress (13, 34, 35). To explore the responses of small GTPase genes in *L. edodes* under different stress conditions, we analyzed the expression profiles of selected *L. edodes* small GTPase genes under abiotic stresses (including low temperature, high temperature, cadmium exposure, and light exposure) and biotic stress caused by *Trichoderma* spp. infection. The qRT-PCR analysis showed that most of the *L. edodes* small GTPase genes exhibited different degrees of responses to these stresses (Fig. 7; Fig. S5) with different response patterns including both positive and negative responses. The members of the subfamily exhibited synergistic responses.

Under heat stress, the expression levels of most small G-protein family genes in *L. edodes* were upregulated. Within the Rho subfamily, *LeRho1*, *LeRac*, *LeCDC42*, *LeMitRho1*, and *LeMitRho2* showed significant upregulation. In the Ras subfamily, *LeRas1* was significantly upregulated, while *LeRhb2* was significantly downregulated. In the Arf subfamily, *LeArf1*, *LeArf2*, *LeArl1*, *LeArl2*, *LeArl3*, *LeGtr1*, and *LeSar1* were significantly upregulated. All Rab subfamily genes were significantly upregulated under heat stress. The Ran subfamily contains only one gene, *LeRan*, which also showed significant upregulation (Fig. 7; Fig. S5A).

Under cold stress, the upregulated genes in the Rho subfamily included *LeRho2*, *LeRho3*, *LeRho4*, *LeRac*, and *LeMitRho2*. In the Ras subfamily, *LeRas2*, *LeRhb1*, *LeRhb2*, and *LeRSR1* were upregulated. For the Arf subfamily, upregulated genes were *LeArf1*, *LeArf2*, *LeArl2*, *LeArl3*, *LeGtr1*, *LeGtr2*, and *LeCin4*, with *LeArl2*, *LeArl3*, and *LeGtr2* showing over 3-fold increases. In the Rab subfamily, *LeYpt31*, *LeYpt32*, *LeSec4*, *LeVPS21*, *LeYpt6*, and *LeTem1* were upregulated. The sole Ran subfamily gene, *LeRan*, exhibited over a 6.5-fold increase under cold stress (Fig. 7; Fig. S5B).

Under cadmium ion (Cd²^+^) stress, all genes in the Rho and Rab subfamilies were upregulated, including *LeRac*, *LeVps21*, and *LeYpt52* over 3-fold increases. In the Ras subfamily, *LeRas1*, *LeRas2*, *LeRhb1*, and *LeRhb2* were upregulated. Some genes in the Arf and Ran subfamilies demonstrated higher fold changes compared to other subfamilies under Cd²^+^ stress. *LeArf1*, *LeArf2*, *LeArl2*, *LeArl3, LeGtr1*, *LeGtr2*, *LeCin4*, and *LeRan1* were significantly upregulated, with *LeArl3*, *LeGtr2*, and *LeRan* exceeding 7-fold increases (Fig. 7; Fig. S5C).

Under *Trichoderma* stress, almost all small GTPase genes in *L. edodes* were significantly upregulated, except for *LeArf1*, *LeArf3*, and *LeSar1* from the Arf family. Among the remaining 31 genes, *LeRho2*, *LeRho3*, *LeMitRho1*, *LeMitRho2*, *LeRhb1*, *LeArf1*, *LeArl1*, and *LeCin4* were upregulated more than 3-fold; *LeArl2* and *LeGtr1* were upregulated over 6-fold; and *LeArl3*, *LeGtr2*, and *LeRan* exhibited upregulation exceeding 40-fold (Fig. 7; Fig. S5D).

When *L. edodes* mycelia were exposed to light, the Rho subfamily genes *LeRho1*, *LeRho2*, *LeRac*, *LeCdc42,* and *LeMitRho1* were significantly upregulated. In the Ras subfamily, *LeRas1*, *LeRhb1*, *LeRhb2,* and *LeRsR1* showed significant upregulation. For the Arf subfamily, *LeArf3* and *LeGtr2* were significantly upregulated, exceeding 8-fold increases. All Rab subfamily genes were significantly upregulated. The Ran subfamily gene *LeRan* exhibited over an 8-fold increase (Fig. 7; Fig. S5E).

### Stress resistance of LeRho1 overexpressed transformants in *L. edodes*

**Fig 8:**
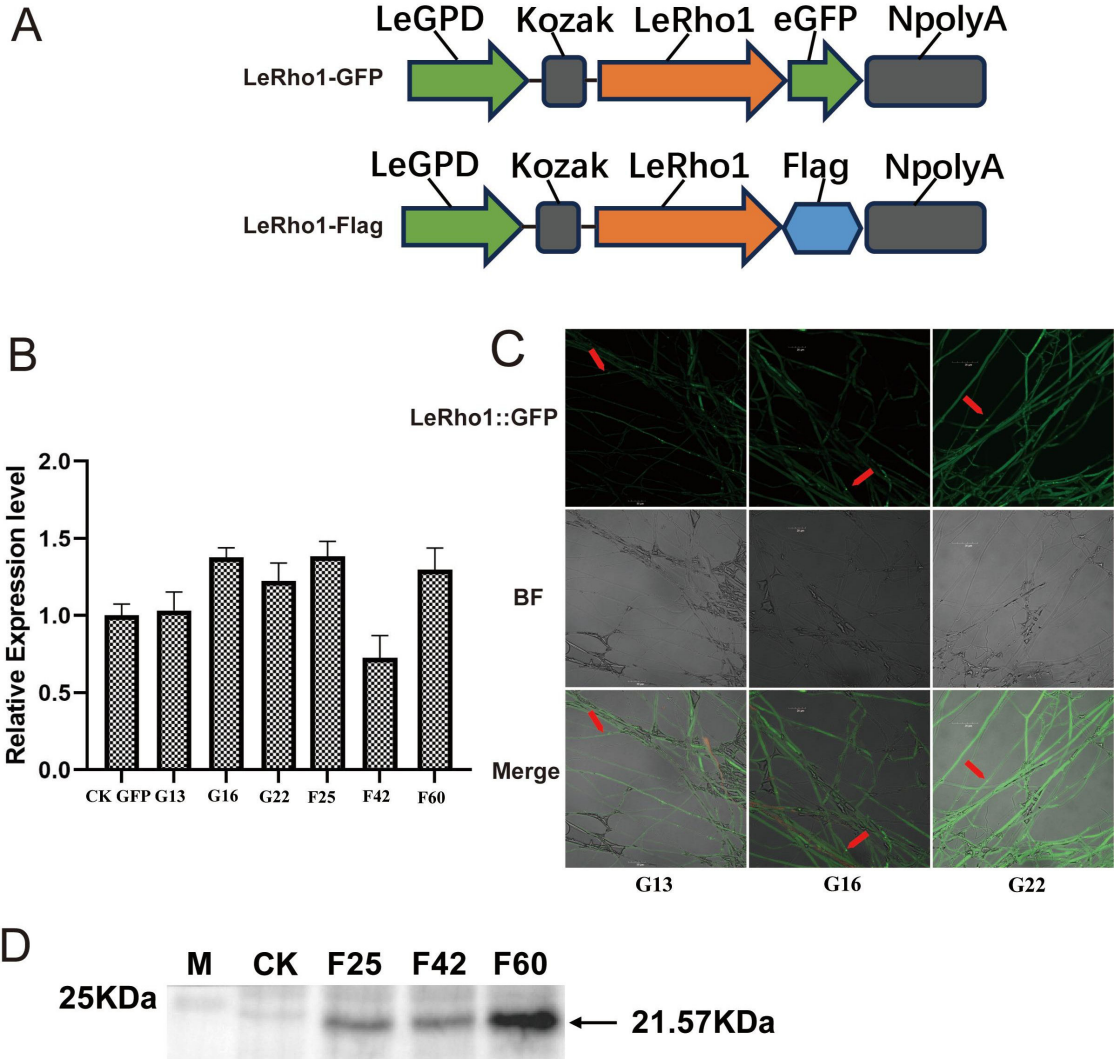
Screening of overexpressed *LeRho1* transformants in *L. edodes*. (**A**) Gene expression vector diagram. (**B**) Relative expression of *LeRho1* gene in transformants of *L. edodes*. (**C**) LeRho1-GFP laser confocal microscopy observation. Red arrows indicate strong GFP signal concentrations. (**D**) The expression level of LeRho1-Flag fusion protein in *L. edodes* mycelium by Anti-Flag WB detection.

To screen for *L. edodes* transformants with high *LeRho1* expression, we constructed two fusion protein expression vectors: LeRho1-GFP and LeRho1-Flag (Fig. 8A). These vectors were engineered with a novel overexpression system incorporating the Kozak sequence and the *LeEF1α* promoter to drive the expression of a hygromycin resistance gene (*hyg*) as the selection marker. This design significantly improved the selection efficiency and transgene expression stability in *L. edodes*. Using *Agrobacterium*-mediated genetic transformation, we successfully obtained multiple transformants. These were rigorously evaluated through qRT-PCR (Fig. 8B), confocal laser scanning microscopy (Fig. 8C), and Western blot analysis (Fig. 8D). The results demonstrated robust expression of *LeRho1* in the YS3334 strain, with GFP-tagged transformants exhibiting exceptionally high fluorescence intensity, surpassing existing edible fungal expression systems. Western blot analysis further confirmed the high-level overexpression of LeRho1 protein in selected transformants.

**Fig 9:**
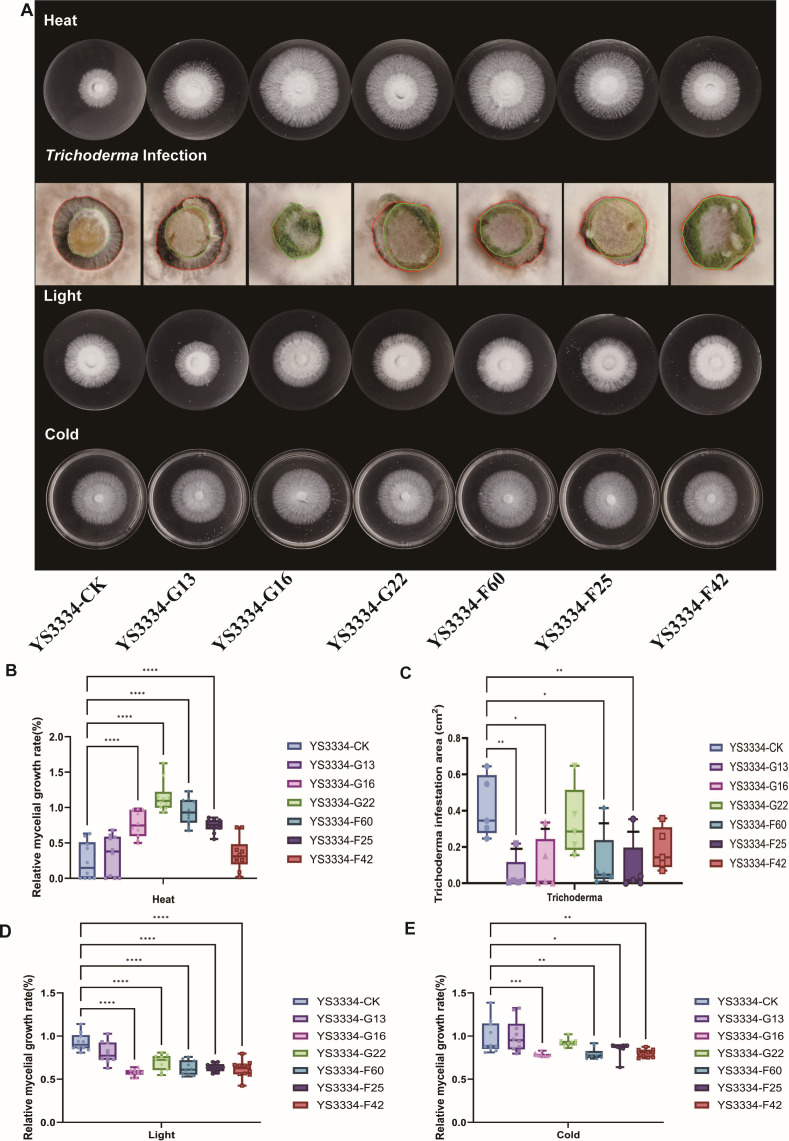
Evaluation of stress resistance in *LeRho1* overexpression transformants of *L. edodes* mycelium. (**A**) The mycelial growth of *L. edodes* overexpression transgenic strain under environmental conditions stress. (**B**) Relative mycelial growth recovery rate in *LeRho1*-overexpressing versus empty vector control strains after heat stress. (**C**) Relative mycelial growth recovery rate in *LeRho1*-overexpressing versus empty vector control strains after *Trichoderma* infection. (**D**) Relative mycelial growth recovery rate in *LeRho1*-overexpressing versus empty vector control strains after light stress. (**E**) Relative mycelial growth recovery rate in *LeRho1*-overexpressing versus empty vector control strains after cold stress (*: *P* ≤ 0.05, **: *P* ≤ 0.01,***: *P* ≤ 0.001,****: *P* ≤ 0.0001).

For phenotypic assays, we selected three transformants from each vector with the highest *LeRho1* expression levels. Under heat stress, the mycelial diameter recovery of *L. edodes* transformants LeRho1-G16, LeRho1-G22, LeRho1-F60, and LeRho1-F25 was significantly greater than that of the control. The average relative mycelium growth rate ranged from 76.7% (LeRho-F25) to 115.2% (LeRho1-G22). (Fig. 9B). In contrast, transformants LeRho1-G13 and LeRho1-F42 showed only marginal improvements, with no statistically significant difference compared to the control.

In confrontation assays with *Trichoderma*, the infection area in transformants LeRho1-G13, LeRho1-G16, LeRho1-F60, and LeRho1-F25 was significantly reduced compared to the control group. Specifically, the infection area in the control group was 0.418 cm^2^, whereas in the *LeRho1* overexpressing transformants, it was reduced to 0.05 cm^2^ (LeRho1-G22)–0.18 cm^2^ (LeRho1-F25) (Fig. 9C), demonstrating a marked enhancement in anti-contamination ability.

Under light exposure, the mycelial diameter recovery of all *L. edodes* transformants, except for LeRho1-G13, was significantly lower than that of the control. The control strain exhibited a relative mycelium growth rate of 93.0%, while the *LeRho1* overexpressing transformants showed relative mycelial growth rate ranging from 57.8% (LeRho-G16) to 69.7% (LeRho1-G22 ) (Fig. 9D), indicating heightened sensitivity to light.

Under cold stress conditions, the mycelial growth rates of *LeRho1* transformants (*L. edodes* strains YS3334-G16, YS3334-F60, YS3334-F25, and YS3334-F42) showed significant decreases compared to the control group YS3334-CK (Fig. 9A and E). These results indicate that overexpression of *LeRho1* leads to slower mycelial growth of the transformants under cold stress.

These results collectively demonstrate that *LeRho1* overexpression in *L. edodes* significantly enhances thermotolerance, anti-contamination ability, light sensitivity, and cold sensitivity. The successful implementation of our novel overexpression system, coupled with the phenotypic alterations observed, underscores the potential of *LeRho1* as a key target for molecular breeding in edible fungi.

## DISCUSSION

Understanding the molecular mechanisms of stress tolerance in edible fungi is essential for developing environmentally resilient cultivars. Small GTPases have emerged as one kind of pivotal regulators governing cellular adaptation to environmental stress across eukaryotes (13). In this study, we identified all 34 small GTPase family members in *L. edodes* and analyzed their phylogenetic relationships within Ascomycetes and Basidiomycetes. By examining the transcriptional expression profiles of these genes under five common stress conditions (heat, cold, light, cadmium exposure, and *Trichoderma* infection), we found that most small GTPase genes exhibited significant stress-responsive expression patterns, indicating their crucial roles in fungal adaptation to both abiotic and biotic stresses. We also developed a novel *L. edodes*-specific Kozak sequence and integrated it into the genetic transformation system, resulting in transgenic strains with improved genetic stability and enhanced fluorescence intensity in LeRho1-GFP transformants. Additionally, among the small GTPase candidates, *LeRho1* was selected for function analysis. The phenotypic assay of *LeRho1* overexpression transformants indicated that LeRho1 is important for regulating thermotolerance, resistance to *Trichoderma* infection, light sensitivity, and cold sensitivity.

The phylogenetic tree of small GTPase proteins from four Ascomycetes and three Basidiomycetes species revealed distinct evolutionary patterns within the family. All 34 *L. edodes* small GTPases clustered into five canonical subfamilies (Rho, Rab, Arf, Ran, and Ras), with homologs from Ascomycetes and Basidiomycetes forming separate branches within each subfamily (Fig. 5). This phylogenetic separation aligns with the evolutionary divergence between these two fungal phyla and suggests that the core functions of small GTPases (e.g., membrane trafficking, cytoskeletal regulation) are conserved across fungal lineages. A novel clade, MitRho, was identified within the Rho subfamily, containing two *L. edodes* members (*LeMiRho1* and *LeMitRho2*) and homologs from *L. bicolor* and *V. volvacea* (Fig. 5). The absence of *MitRho* in Ascomycetes (only *A. fumigatus* has one *MitRho*) suggests that this lineage-specific expansion may contribute to Basidiomycetes’ unique stress response mechanisms. This lineage-specific expansion of the *MitRho* subfamily in Basidiomycetes, along with the hypothesis that it may function in mitochondrial stress adaptation, highlights a novel direction for investigating stress resistance mechanisms in edible fungi. Our qRT-PCR results demonstrated that *LeMitRho1* and *LeMitRho2* exhibited varying degrees of upregulation under all five stress conditions examined in this study. The most pronounced upregulation was observed specifically under high-temperature stress and *Trichoderma* infection, lending support to the aforementioned hypothesis.

The edible mushroom industry faces severe challenges from climate change. Enhancing the stress tolerance of high-economic-value species such as *L. edodes*, *Pleurotus ostreatus,* and *Ganoderma lucidum* is a core requirement for the sustainable development of the industry. Research indicates that in *G. lucidum*, nitric oxide (NO) regulates heat-stress-induced ganoderic acid synthesis by inhibiting mitochondrial succinate dehydrogenase (34). Concurrently, intracellular Ca^2+^ and ROS signaling coordinates hyphal growth and secondary metabolism through the activation of heat shock proteins (HSPs) (35). Omics data from *P. ostreatus* and *L. edodes* under high-temperature stress (40°C) further reveal that heat stress triggers core carbon metabolic reprogramming. This is accompanied by mitochondrial dysfunction and glycolysis-dependent lactate accumulation, ultimately inhibiting hyphal growth, while the upregulation of HSPs constitutes a critical stress response strategy (9, 36). Notably, small GTPases act as signaling hubs that integrate Ca^2+^, ROS, and metabolic stress signals, directly regulating cytoskeletal dynamics, vesicle trafficking, and HSP expression (37, 38). Consequently, dissecting the small GTPase signaling network represents a crucial breakthrough point for elucidating the molecular mechanisms of stress tolerance in edible fungi.

However, current research remains largely confined to omics-based correlative analyses, lacking direct experimental validation of gene function. This study overcame this limitation by optimizing the genetic transformation system for *L. edodes*, successfully establishing a fluorescent fusion protein expression system. Live-cell imaging using this system demonstrated that the fluorescence signal intensity and localization resolution are sufficient for resolving the spatiotemporal dynamics of target proteins within the complex hyphal networks of filamentous fungi. Leveraging this system, we phenotypically revealed the functional versatility of *L. edodes* small GTPases (e.g., LeRho1) in mediating responses to heat stress, defense against *Trichoderma* infection, and light perception. Intriguingly, *LeRho1* mRNA levels remained stable despite protein overexpression (Fig. 8B/D); this underscores the necessity of protein-level analysis in studying regulatory gene function. Measuring the fluorescence intensity of fusion proteins provides an efficient and rapid method for assessing protein expression levels. This work not only provides functional evidence for understanding stress tolerance mechanisms in edible fungi but also establishes a widely applicable molecular toolkit. It lays a solid foundation for the future systematic screening of conserved small GTPase targets and in-depth mechanistic dissection.

The Rho1 in *S. cerevisiae* is a homolog of the animal RhoA protein (39), but its function exhibits unique features: Rho1 in *S. cerevisiae* not only functions as a small GTPase molecular switch but also acts as a subunit of β-1,3-glucan synthase (Fks1/2) involved in cell wall synthesis (16). Since the function of the Rho1 protein, as a molecular switch, is primarily achieved by regulating the ratio of the active Rho1-GTP state to the inactive Rho1-GDP state, this regulation is not affected by an increase in protein expression level. Therefore, in this study, overexpression of *LeRho1* may not have affected signal transduction by altering the ratio of the active state of Rho1 (LeRho1-GTP/LeRho1-GDP), but rather—since LeRho1 serves as a subunit of Fks1/2—through elevated LeRho1 protein levels enhancing Fks1/2 activity, thereby strengthening the structure of the *L. edodes* cell wall and ultimately improving its stress resistance (16, 40).

In model fungi, the function of *Rho1* has been relatively well studied. For example, in yeast, the Rho1 protein participates in signaling pathways related to oxidative stress, osmotic stress, and cell wall integrity; it primarily maintains cell wall structural integrity by regulating the activity of β-1,3-glucan synthase (16). Similarly, in *Grifola frondosa*, deletion of the Rho1 gene leads to a significant reduction in β−1,3-glucan content in the cell wall, thereby compromising cell wall structure (41). These studies indicate that the Rho1 protein plays a critical role in maintaining the cell wall structure of fungi. As the primary barrier for fungi in contact with the external environment, the cell wall plays an important role in stress resistance. However, current research on the cell wall of edible fungi under stress conditions remains relatively scarce. Therefore, future research should focus on the cell wall, delving into its mechanisms during stress responses, to further elucidate the molecular regulatory network of stress resistance in edible fungi.

This work bridges a critical knowledge gap in Agaric cell wall integrity regulation and provides molecular tools for developing stress-resistant edible fungi, addressing significant agricultural challenges posed by rising temperatures and biotic threats.

## Data Availability

The data sets generated and/or analyzed during the current study are available in the Gene Expression Omnibus database under accession no. GSE219161. Requests for material should be made to the corresponding author.

## SUPPLEMENTAL MATERIAL

Supplemental material aem.01967-25-s0001.pdf Tables S1 to S3; Fig. S1 to S5.

ASM does not own the copyrights to Supplemental Material that may be linked to, or accessed through, an article. The authors have granted ASM a non-exclusive, world-wide license to publish the Supplemental Material files. Please contact the corresponding author directly for reuse.
