# Supplementary material for: Genome-wide identification of small GTPase gene family members in Lentinula edodes and the roles of LeRho1 in biotic and abiotic stress responses
Source: Appl Environ Microbiol. 2026 Mar 18;92(4):e01967-25. doi: 10.1128/aem.01967-25 (PMC13101504; doi:10.1128/aem.01967-25)
Supplement: Supplemental material — Tables S1 to S3; Fig. S1 to S5. [file aem.01967-25-s0001.pdf]

Query	From	To	E-Value	Bitscore	Short name
Q#1->LE01Gene06942.t1	10	173	2.37E-96	277.082	RAB
Q#2 - >LE01Gene10628.t1	6	175	6.23E-90	260.903	RAB
Q#3 - >LE01Gene13471.t1	19	175	2.65E-80	236.636	RAB
Q#4 - >LE01Gene08865.t1	3	177	2.30E-133	370.358	Cdc42
Q#5 - >LE01Gene00143.t1	7	168	2.80E-75	223.924	RAB
Q#6 - >LE01Gene03440.t1	3	176	2.05E-132	368.371	Rac1_like
Q#7 - >LE01Gene01429.t1	6	178	2.13E-126	352.302	ARF
Q#8 - >LE01Gene05769.t1	5	179	4.25E-126	352.116	RhoA_like
Q#9 - >LE01Gene01831.t1	5	178	1.20E-118	333.042	ARF
Q#10 - >LE01Gene08878.t1	11	174	2.23E-109	310.209	RAB
Q#11 - >LE01Gene10526.t1	12	177	8.09E-88	256.281	RAB
Q#12 - >LE01Gene00998.t1	22	167	1.58E-87	254.075	RHO
Q#13 - >LE01Gene05414.t1	21	182	1.55E-78	231.734	ARF
Q#14 - >LE01Gene05585.t1	10	201	6.01E-95	274.77	RAB
Q#15 - >LE01Gene14437.t1	15	179	2.68E-34	118.871	ARF
Q#16 - >LE01Gene13007.t1	10	203	3.16E-79	235.48	RAB
Q#17 - >LE01Gene13143.t1	23	173	2.81E-76	226.627	Ras
Q#18 - >LE01Gene07233.t1	16	212	2.41E-75	225.857	Ras
Q#18 - >LE01Gene07233.t1	15	211	3.44E-73	220.457	RAB
Q#19 - >LE01Gene03508.t1	47	217	2.37E-92	268.328	RHO
Q#20 - >LE01Gene01682.t1	9	189	8.46E-95	272.967	RheB
Q#21 - >LE01Gene04214.t1	11	182	1.92E-87	255.616	RHO
Q#22 - >LE01Gene01043.t1	5	154	4.24E-51	161.243	ARF
Q#23 - >LE01Gene10327.t1	16	166	1.81E-53	167.692	Ras
Q#24 - >LE01Gene13383.t1	19	107	6.81E-22	92.5711	RAB
Q#25 - >LE01Gene09341.t1	2	189	4.99E-125	350.04	Sarl
Q#26 - >LE01Gene04244.t1	38	205	1.34E-32	115.683	RAB
Q#27 - >LE01Gene10676.t1	5	165	2.48E-69	208.138	Ras
Q#28 - >LE01Gene10475.t1	37	185	2.47E-44	144.965	Ras
Q#29 - >LE01Gene04939.t1	17	192	2.06E-17	79.195	ARF
Q#30 - >LE01Gene09510.t1	3	171	5.60E-95	288.852	Miro1
Q#31 - >LE01Gene 10025.t1	4	208	6.56E-101	296.415	Gtrl_RagA
Q#32 - >LE01Gene00894.t1	1	127	7.11E-82	238.373	RAN
Q#33 - >LE01Gene04039.t1	5	78	4.72E-17	73.417	ARF
Q#34 - >LE01Gene01305.t1	16	274	8.41E-55	184.707	Gtrl_RagA

TableS1. *L. edodes* small GTPase conserved domin analysis by the NCBI Batch CD - Search tools.

Primer name	Primer sequence(5' to 3')
HR-Rho1-GFP_F	gcccttgctcaccatGGATCCGACGACAACACACTTGCTACCCT
HR-Rho1-GFP_R	gcccccggggatggcggatccATGTCTGAAATTAGGAGGAAACTCG
pOE-Rho1-Flag_F	GGGAAATTCGAGCTCGAATTcTTAGACGACAACACACTTGCTACCC
pPOE-Rho1-Flag_R	gcccccggggatggcggatccATGGATTACAAGGACGA CGATGACAAGTCTGAAATTAGGAGGAAACTCGTTATC
DL_LE01Gene06942.t1_F	ACCGAGGAGCTATGGGCA
DL_LE01Gene06942.t1_R	CCGAGGCATGCTGTTCGA
DL_LE01Gene10628.t1_F	GTGGACGACCGGCTTGTT
DL_LE01Gene10628.t1_R	TGGCGCTGTTTACGTCGT
DL_LE01Gene13471.t1_F	ACATCGGCAAAGGCTGGT
DL_LE01Gene13471.t1_R	AGCCTCTGGGACCTCGTT
DL_LE01Gene08865.t1_F	AACCAGTGATGGGCGAGC
DL_LE01Gene08865.t1_R	AGGTTCAAGGGCAGCGAC
DL_LE01Gene00143.t1_F	TGCAAAGACTGGCGAGGG
DL_LE01Gene00143.t1_R	GCGACCTGCACCAGTTCT
DL_LE01Gene03440.t1_F	GAAGCTGAGGGACCGTCG
DL_LE01Gene03440.t1_R	CACGGATTGCCTCGTCGA
DL_LE01Gene01429.t1_F	CCTTGACGCCGCTGGTAA
DL_LE01Gene01429.t1_R	CTTGCCCTCCGACATCCC
DL_LE01Gene05769.t1_F	GCTCAGCCAAGTCTGGGG
DL_LE01Gene05769.t1_R	TTCTTCTTGCCTGGGCGG
DL_LE01Gene01831.t1_F	CTGGAGCAATGTCCCCGG
DL_LE01Gene01831.t1_R	CCGGTTGTGGCACAGCTA
DL_LE01Gene08878.t1_F	CTACGGGATCACGCCGAC
DL_LE01Gene08878.t1_R	CGTCCAGCGCTGATGTCT
DL_LE01Gene10526.t1_F	ATGGCACCAACCGGATCG
DL_LE01Gene10526.t1_R	CCACACCAATCGTTGCGC
DL_LE01Gene00998.t1_F	ACCGCTCAGCTACCCAGA
DL_LE01Gene00998.t1_R	TCCGACCAGGATGAGGGG
DL_LE01Gene05414.t1_F	AGGCCAGTCCAGTATCCG
DL_LE01Gene05414.t1_R	TAGACGGGCTTGGTCGGA
DL_LE01Gene05585.t1_F	TCCTGGAGACATCGGCCA
DL_LE01Gene05585.t1_R	GGTGTGGACCCCATTCGG
DL_LE01Gene14437.t1_F	TCCAGGCTTCCGGGAAGA
DL_LE01Gene14437.t1_R	TCCCCTTCCGGACCTTCC
DL_LE01Gene13007.t1_F	ATCAATCCAACGCCGCCA
DL_LE01Gene13007.t1_R	CCTCGCAGCCTGTTCGAA
DL_LE01Gene13143.t1_F	CAACGTCGACCAGGCCTT
DL_LE01Gene13143.t1_R	CCTCCTCCCCCTCCAGTC
DL_LE01Gene07233.t1_F	ACGCAGCTTCCGGTTCAA
DL_LE01Gene07233.t1_R	GTCGGCTCCTTGTTGGCT
DL_LE01Gene03508.t1_F	ACGACTGCAGCGATACGG
DL_LE01Gene03508.t1_R	GCTTCGAGCTGCACTCCA
DL_LE01Gene01682.t1_F	ACGGCGAGAAGTTGGCTC

DL_LE01Gene01682.t1_R	CTTCGCCGGTCTGTGGAG
DL_LE01Gene04214.t1_F	AACACGGACGGTCGTCAC
DL_LE01Gene04214.t1_R	GCACACAACGCAGCACTT
DL_LE01Gene01043.t1_F	AGCGGACCCTACGACCAT
DL_LE01Gene01043.t1_R	CGCATTCGGTCCCCAGAG
DL_LE01Gene10327.t1_F	TCTGACCCAGGAGGCGAG
DL_LE01Gene10327.t1_R	TTGCTCCAGCTTCGCCTT
DL_LE01Gene13383.t1_F	GGGAGGATTCCGGGGACT
DL_LE01Gene13383.t1_R	AATCCCGCTGCCATGTCC
DL_LE01Gene09341.t1_F	GCAAAGTTCCGCTCACCG
DL_LE01Gene09341.t1_R	AACCGTCCCCGTAACCCT
DL_LE01Gene04244.t1_F	AGACCATCTCAGTGCGGC
DL_LE01Gene04244.t1_R	AATAGGATGGCCACCGCG
DL_LE01Gene10676.t1_F	GGACGAAGGACAGGCACT
DL_LE01Gene10676.t1_R	ACATTGCGTGTCCGTCGT
DL_LE01Gene10475.t1_F	CAACCATTGAGCGCGCAT
DL_LE01Gene10475.t1_R	CGTAGCCGTGGATGCCAA
DL_LE01Gene04939.t1_F	AACCGGGTGGGGCTCATA
DL_LE01Gene04939.t1_R	ATGACGGCTTTGCGTCCA
DL_LE01Gene09510.t1_F	ACCAGGCAATCCTTGGGC
DL_LE01Gene09510.t1_R	CAGCCCTGCAGAGTGACC
DL_LE01Gene10025.t1_F	CGCCTCCACATCCTCGAC
DL_LE01Gene10025.t1_R	AGTTCGGAGGTGCGTTCG
DL_LE01Gene00894.t1_F	GCGAGGAAGTTGGTCGGT
DL_LE01Gene00894.t1_R	GGGAACAGCCTCAGCCTG
DL_LE01Gene04039.t1_F	TATTGACGCCGACGACCG
DL_LE01Gene04039.t1_R	TAGGAGGAGGGGCACACC
DL_LE01Gene01305.t1_F	TTCACCCGTCGACACTGC
DL_LE01Gene01305.t1_R	CCGTCGTCGTCGTACAGG
DL_EF1A_F	CCGTCAACATGCCCTGGT
DL_EF1A_R	AGGACGGACAGGGGTCTC
DL_PMA_F	GGTGCGTTCTGTGTACCCCA
DL_PMA_R	TCGCGAACTTGATCCAGTCGA
DL_28S_F	TGGCTCTAAGGGTTGGGTGC
DL_28S_R	CCCGAAGAGCAGCCAAAGTC

Table S2. Sequence information of primers used in this study

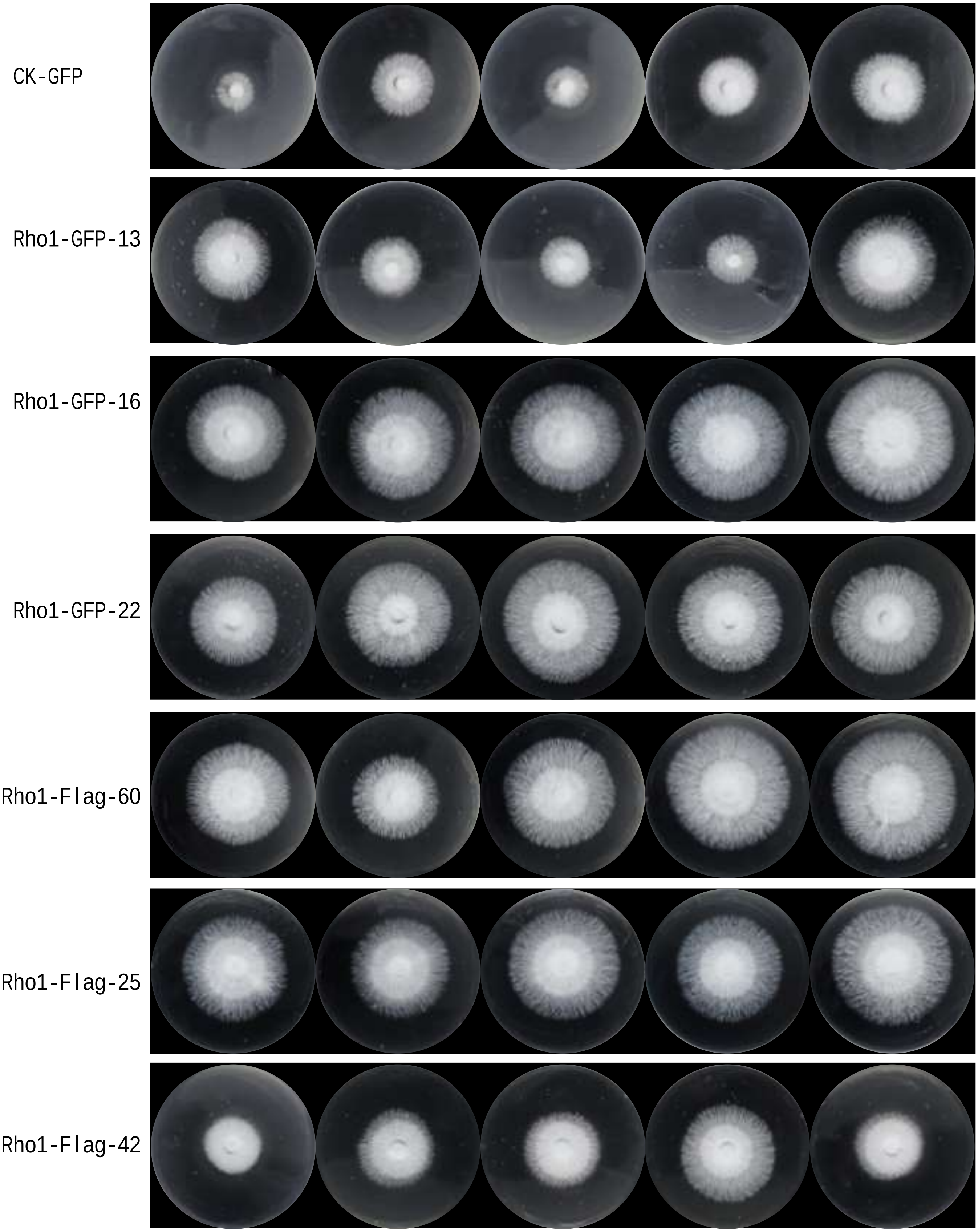

Figure S1 Recovery of *L. edodes* mycelium after heat stress

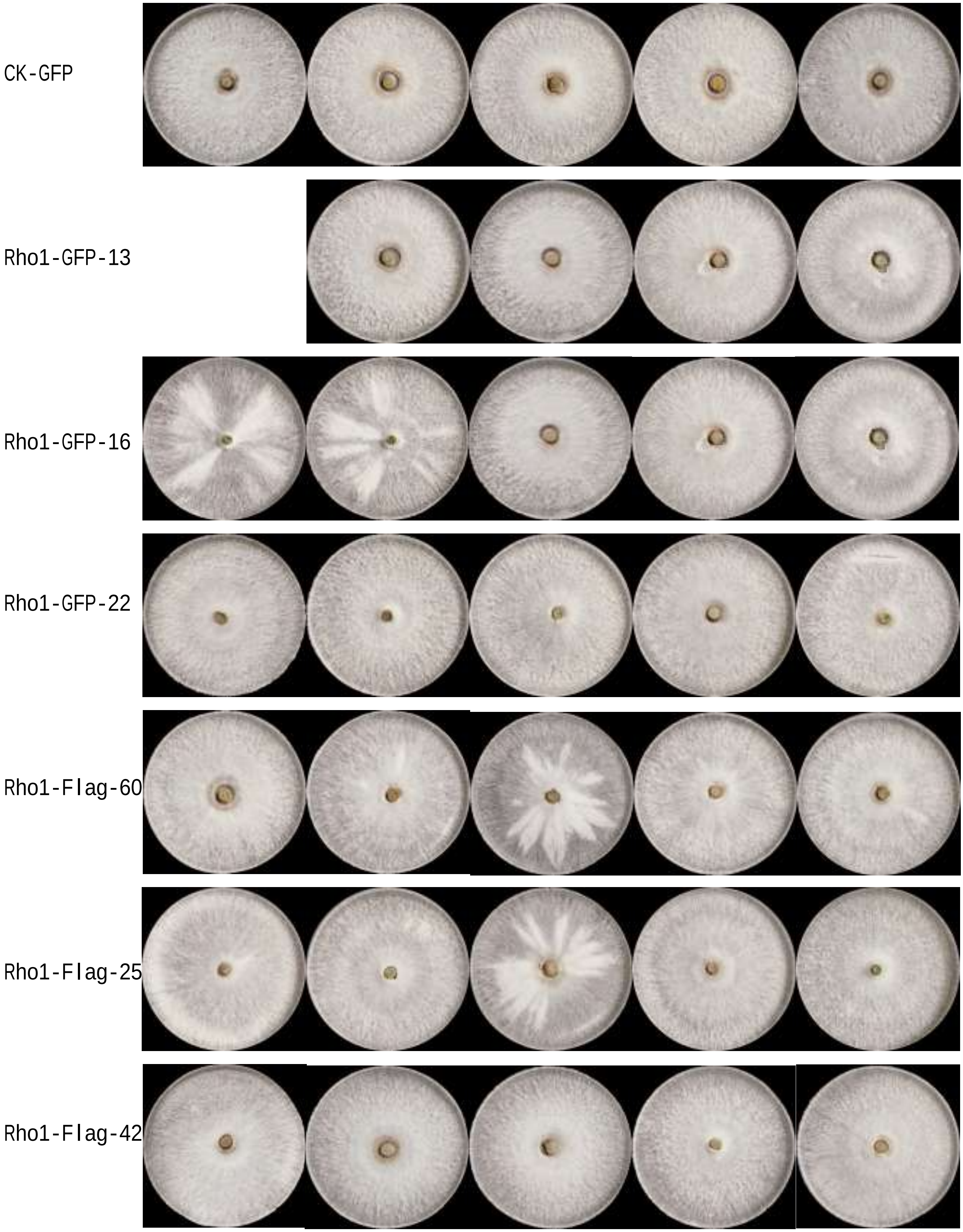

Figure S2 *Trichoderma* infects mycelium of *L. edodes*.

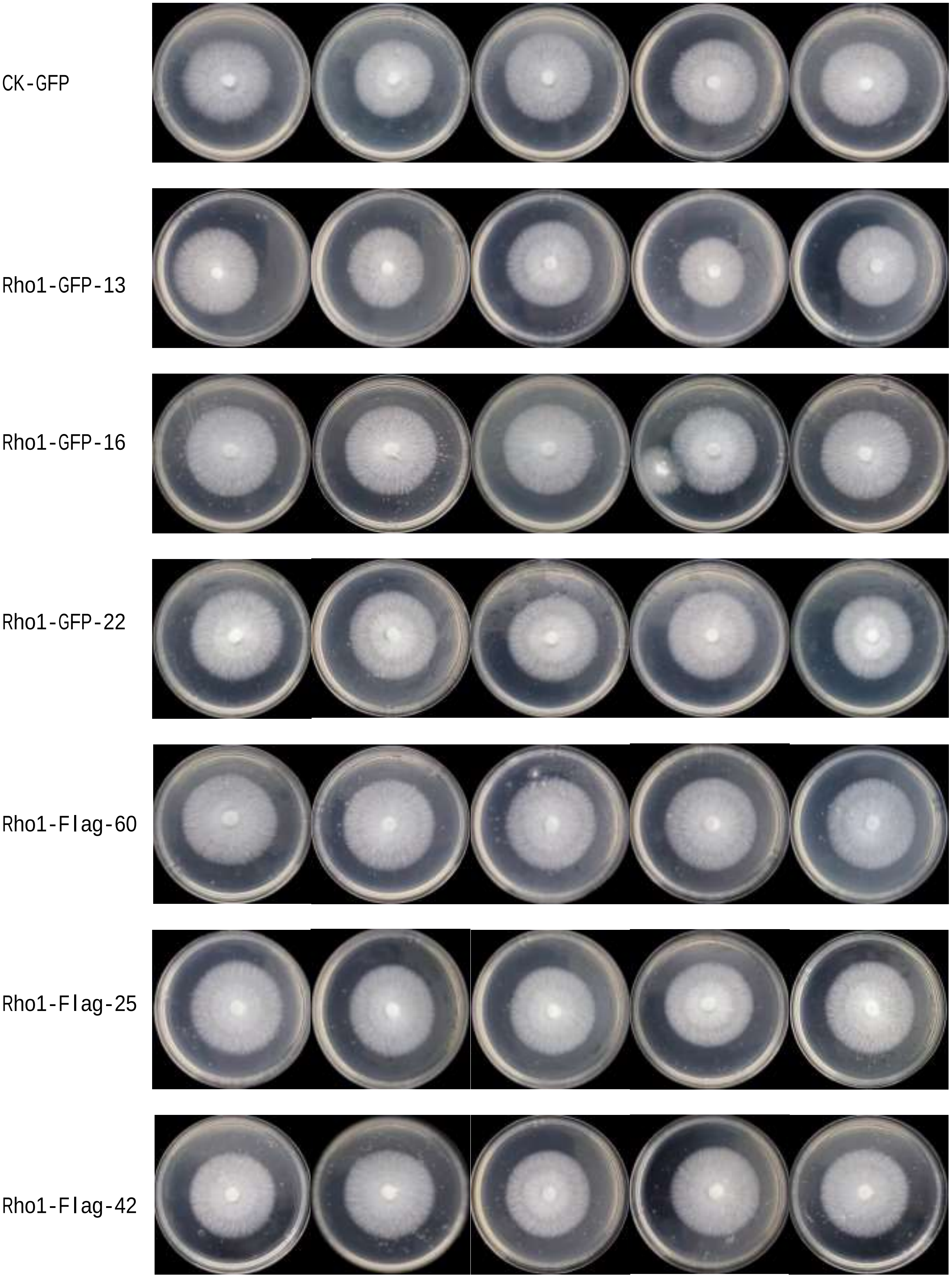

Figure S3 Recovery of *L. edodes* mushroom mycelium after cold stress.

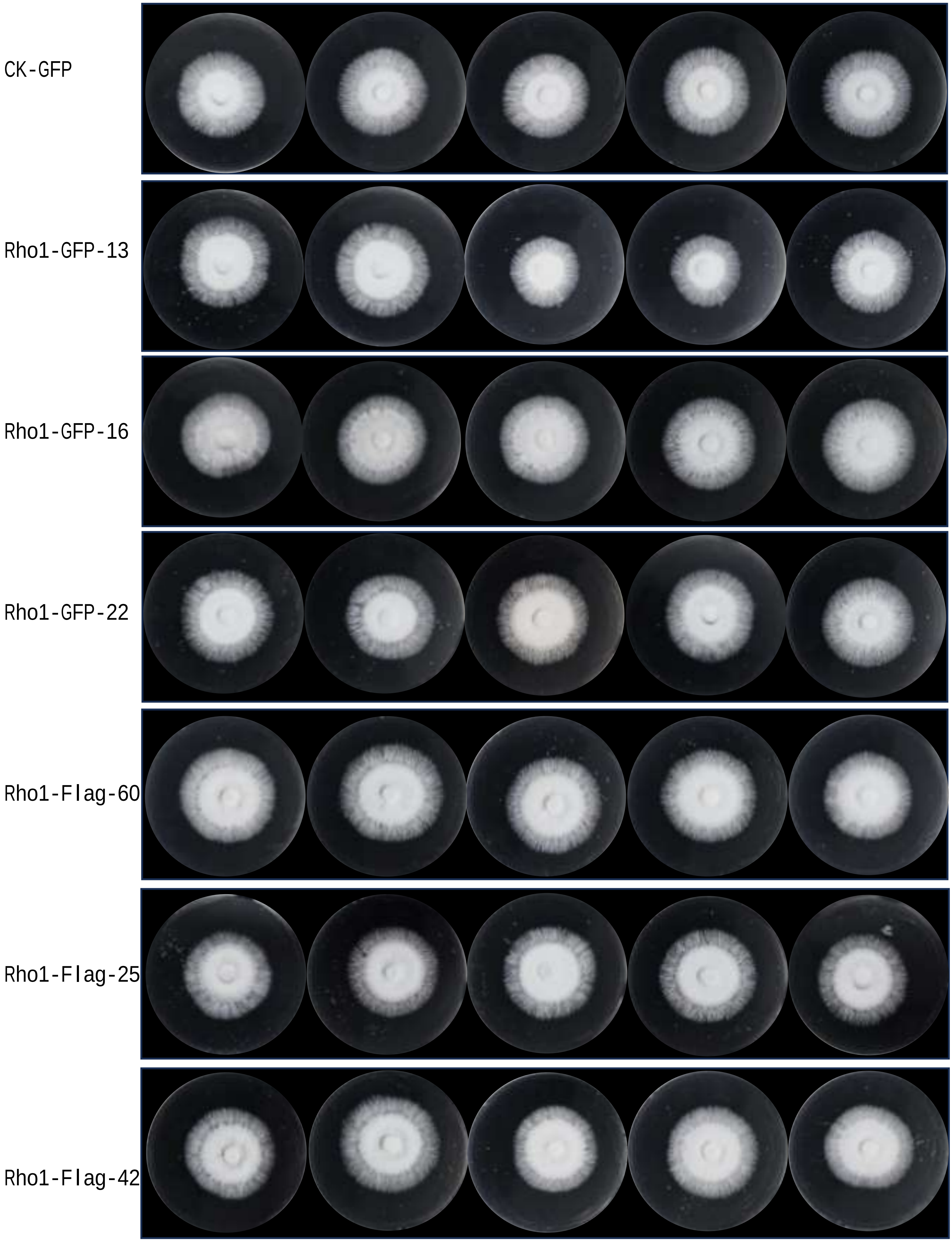

Figure S4 Recovery of *L.edodes* mycelium resumes after exposure to light

Table S3 Mycelial Relative Growth Rate and Computational Intermediates

Heat: (mycelium diameter (cm))	Sample 1				Sample 2				Sample 3				Sample 4				Sample 5			
	D1A	D2A	D1B	D2B	D1A	D2A	D1B	D2B	D1A	D2A	D1B	D2B	D1A	D2A	D1B	D2B	D1A	D2A	D1B	D2B
YS3334-CK	2.90	3.55	2.60	3.70	2.50	3.70	2.80	3.00	2.90	2.90	2.85	2.85	2.70	2.70	2.80	2.80	1.90	3.10	2.00	3.20
YS3334-G13	3.20	5.00	3.10	4.90	2.75	2.75	2.80	2.90	2.85	2.85	2.70	2.70	2.40	3.25	2.35	3.30	2.50	4.20	2.55	4.10
YS3334-G16	3.15	6.25	3.15	6.20	3.05	6.00	3.00	5.75	3.20	5.40	3.15	5.40	3.15	5.60	3.20	5.15	3.10	4.65	3.10	4.80
YS3334-G22	2.55	5.40	2.70	5.40	2.70	5.35	2.80	5.40	2.75	5.65	2.85	5.90	2.10	5.15	2.00	5.25	2.05	4.40	2.10	4.50
YS3334-F60	2.95	6.25	3.00	6.25	2.85	6.35	2.90	6.10	2.80	5.50	2.85	5.40	2.60	4.35	2.55	4.40	2.80	5.05	2.75	5.10
YS3334-F25	3.15	5.85	3.25	5.95	3.05	5.30	3.05	5.40	3.15	5.70	3.05	5.55	3.15	4.90	3.15	5.15	3.10	5.30	3.15	5.40
YS3334-F42	3.00	3.70	2.95	3.80	2.85	4.90	2.85	4.90	2.85	4.00	3.00	4.00	3.10	3.90	2.90	4.00	2.90	3.15	2.90	2.95

Heat: Mycelial regrowth diameter(cm)	Sample 1		Sample 2		Sample 3		Sample 4		Sample 5	
	DA	DB	DA	DB	DA	DB	DA	DB	DA	DB
YS3334-CK	0.65	1.10	1.20	0.20	0.00	0.00	0.00	0.00	1.20	1.20
YS3334-G13	1.80	1.80	0.00	0.10	0.00	0.00	0.85	0.95	1.70	1.55
YS3334-G16	3.10	3.05	2.95	2.75	2.20	2.25	2.45	1.95	1.55	1.70
YS3334-G22	2.85	2.70	2.65	2.60	2.90	3.05	3.05	3.25	2.35	2.40
YS3334-F60	3.30	3.25	3.50	3.20	2.70	2.55	1.75	1.85	2.25	2.35
YS3334-F25	2.70	2.70	2.25	2.35	2.55	2.50	1.75	2.00	2.20	2.25
YS3334-F42	0.70	0.85	2.05	2.05	1.15	1.00	0.80	1.10	0.25	0.05

Heat: Relative growth rate of mycelia	Sample 1		Sample 2		Sample 3		Sample 4		Sample 5		Mean	SD	Range	Median
	RA	RB	RA	RB	RA	RB	RA	RB	RA	RB				
YS3334-CK	22.41%	42.31%	48.00%	7.14%	0.00%	0.00%	0.00%	0.00%	63.16%	60.00%	24.30%	26.52%	0.00% ~ 63.16%	14.78%
YS3334-G13	56.25%	58.06%	0.00%	3.57%	0.00%	0.00%	35.42%	40.43%	68.00%	60.78%	32.25%	28.59%	0.00%~68.00%	37.93%
YS3334-G16	98.41%	96.83%	96.72%	91.67%	68.75%	71.43%	77.78%	60.94%	50.00%	54.84%	76.74%	18.36%	50.00%~98.41%	74.61%
YS3334-G22	111.76%	100.00%	98.15%	92.86%	105.45%	107.02%	145.24%	162.50%	114.63%	114.29%	115.19%	21.95%	92.86%~162.50%	109.39%
YS3334-F60	111.86%	108.33%	122.81%	110.34%	96.43%	89.47%	67.31%	72.55%	80.36%	85.45%	94.49%	18.49%	67.31%~122.81%	92.95%
YS3334-F25	85.71%	83.08%	73.77%	77.05%	80.95%	81.97%	55.56%	63.49%	70.97%	71.43%	74.40%	9.46%	55.56%~85.71%	75.41%
YS3334-F42	23.33%	28.81%	71.93%	71.93%	40.35%	33.33%	25.81%	37.93%	8.62%	1.72%	34.38%	23.16%	1.72~71.93%	31.07%

Cold: (mycelium diameter (cm))	Sample 1				Sample 2				Sample 3				Sample 4				Sample 5			
	D1A	D2A	D1B	D2B	D1A	D2A	D1B	D2B	D1A	D2A	D1B	D2B	D1A	D2A	D1B	D2B	D1A	D2A	D1B	D2B
YS3334-CK	2.55	5.55	2.20	5.25	2.40	4.90	2.20	4.70	2.85	5.35	2.90	5.45	2.95	5.40	2.80	5.30	2.90	5.25	2.80	5.20
YS3334-G13	2.70	5.30	2.85	5.35	2.25	4.70	2.45	4.95	2.90	5.30	2.95	5.30	1.85	4.30	1.80	4.15	2.50	4.85	2.70	5.00
YS3334-G16	3.10	5.60	3.15	5.55	3.15	5.60	3.15	5.55	3.10	5.50	3.10	5.50	2.95	5.40	3.00	5.35	3.10	5.50	3.05	5.45
YS3334-G22	2.95	5.65	2.85	5.50	2.85	5.45	2.70	5.25	2.90	5.40	2.80	5.35	2.75	5.35	2.85	5.40	2.40	4.85	2.65	5.00
YS3334-F60	3.10	5.85	3.00	5.75	3.20	5.65	3.10	5.60	3.25	5.65	3.20	5.75	3.20	5.60	3.15	5.60	3.25	5.65	3.05	5.40
YS3334-F25	3.10	5.75	3.05	5.75	2.80	5.30	2.85	5.40	2.75	5.20	2.80	5.25	2.85	5.35	2.80	5.30	3.00	5.60	3.05	5.00
YS3334-F42	2.85	5.25	2.90	5.25	3.00	5.25	2.95	5.35	2.90	5.25	2.80	5.25	2.90	5.35	3.05	5.40	3.10	5.40	3.05	5.35

Cold: Mycelial regrowth diameter(cm)	Sample 1		Sample 2		Sample 3		Sample 4		Sample 5	
	DA	DB	DA	DB	DA	DB	DA	DB	DA	DB
YS3334-CK	3.00	3.05	2.50	2.50	2.50	2.55	2.45	2.50	2.35	2.40
YS3334-G13	2.60	2.50	2.45	2.50	2.40	2.35	2.45	2.35	2.35	2.30
YS3334-G16	2.50	2.40	2.45	2.40	2.40	2.40	2.45	2.35	2.40	2.40
YS3334-G22	2.70	2.65	2.60	2.55	2.50	2.55	2.60	2.55	2.45	2.35
YS3334-F60	2.75	2.75	2.45	2.50	2.40	2.55	2.40	2.45	2.40	2.35
YS3334-F25	2.65	2.70	2.50	2.55	2.45	2.45	2.50	2.50	2.60	1.95
YS3334-F42	2.40	2.35	2.25	2.40	2.35	2.45	2.45	2.35	2.30	2.30

Cold: Relative growth rate of mycelia	Sample 1		Sample 2		Sample 3		Sample 4		Sample 5		Mean	SD	Range	Median
	RA	RB	RA	RB	RA	RB	RA	RB	RA	RB				
YS3334-CK	117.65%	138.64%	104.17%	113.64%	87.72%	87.93%	83.05%	89.29%	81.03%	85.71%	98.88%	19.02%	81.0.%~138.64%	88.61%
YS3334-G13	96.30%	87.72%	108.89%	102.04%	82.76%	79.66%	132.43%	130.56%	94.00%	85.19%	99.95%	18.86%	79.66%~132.43%	95.15%
YS3334-G16	80.65%	76.19%	77.78%	76.19%	77.42%	77.42%	83.05%	78.33%	77.42%	78.69%	78.31%	2.10%	76.19%~83.05%	77.60%
YS3334-G22	91.53%	92.98%	91.23%	94.44%	86.21%	91.07%	94.55%	89.47%	102.08%	88.68%	92.22%	4.31%	86.21%~102.08%	91.38%
YS3334-F60	88.71%	91.67%	76.56%	80.65%	73.85%	79.69%	75.00%	77.78%	73.85%	77.05%	79.48%	6.11%	73.85%~91.67%	77.42%
YS3334-F25	85.48%	88.52%	89.29%	89.47%	89.09%	87.50%	87.72%	89.29%	86.67%	63.93%	85.70%	7.76%	63.93%~89.47%	88.12%
YS3334-F42	84.21%	81.03%	75.00%	81.36%	81.03%	87.50%	84.48%	77.05%	74.19%	75.41%	80.13%	4.55%	74.19%~87.50%	81.03%

Cold: (mycelium diameter (cm))	Sample 1				Sample 2				Sample 3				Sample 4				Sample 5			
	D1A	D2A	D1B	D2B	D1A	D2A	D1B	D2B	D1A	D2A	D1B	D2B	D1A	D2A	D1B	D2B	D1A	D2A	D1B	D2B
YS3334-CK	2.30	4.60	2.15	4.60	2.55	4.75	2.50	4.55	2.35	4.55	2.20	4.50	2.45	4.75	2.55	4.75	2.55	4.75	2.60	4.70
YS3334-G13	2.70	4.85	2.80	4.90	2.95	5.05	2.85	4.95	3.10	5.05	2.95	5.10	1.85	3.75	1.95	3.75	2.30	4.45	2.35	4.30
YS3334-G16	3.10	4.70	2.85	4.55	3.10	4.70	3.10	4.90	2.95	4.75	3.00	4.75	3.05	4.90	3.10	4.85	3.05	5.00	3.10	4.85
YS3334-G22	2.80	4.90	2.70	4.80	2.55	4.50	2.70	4.65	3.10	4.80	3.05	4.85	2.95	4.75	2.85	4.75	2.75	4.75	2.60	4.70
YS3334-F60	2.90	5.10	3.00	5.00	3.15	5.40	3.05	5.30	3.15	5.10	3.15	5.05	3.15	4.90	3.20	4.90	3.05	4.70	3.00	4.70
YS3334-F25	2.90	4.70	2.90	4.75	3.10	4.85	3.10	4.95	3.00	5.00	3.05	5.00	3.00	5.10	3.00	5.10	3.00	4.90	3.00	4.75
YS3334-F42	2.95	4.90	2.95	4.70	3.05	4.35	2.95	5.30	2.95	4.60	3.00	4.70	2.90	4.90	3.00	5.00	2.90	4.50	2.95	4.90

Light: Mycelial regrowth diameter(cm)	Sample 1		Sample 2		Sample 3		Sample 4		Sample 5	
	DA	DB	DA	DB	DA	DB	DA	DB	DA	DB
YS3334-CK	2.30	2.45	2.20	2.10	2.20	2.30	2.30	2.20	2.20	2.10
YS3334-G13	2.15	2.10	2.10	2.10	1.95	2.15	1.90	1.80	2.15	1.95
YS3334-G16	1.60	1.70	1.60	1.80	1.80	1.75	1.85	1.75	1.95	1.75
YS3334-G22	2.10	2.10	1.95	1.95	1.70	1.80	1.80	1.90	2.00	2.10
YS3334-F60	2.20	2.00	2.25	2.25	1.95	1.90	1.75	1.70	1.65	1.70
YS3334-F25	1.80	1.85	1.75	1.85	2.00	1.95	2.10	2.10	1.90	1.75
YS3334-F42	1.95	1.75	1.30	2.35	1.65	1.70	2.00	2.00	1.60	1.95

Light: Relative growth rate of mycelia	Sample 1		Sample 2		Sample 3		Sample 4		Sample 5		Mean	SD	Range	Median
	RA	RB	RA	RB	RA	RB	RA	RB	RA	RB				
YS3334-CK	100.00%	113.95%	86.27%	84.00%	93.62%	104.55%	93.88%	86.27%	86.27%	80.77%	92.96%	10.48%	80.77%~113.95%	89.95%
YS3334-G13	79.63%	75.00%	71.19%	73.68%	62.90%	72.88%	102.70%	92.31%	93.48%	82.98%	80.68%	12.20%	62.90%~102.70%	77.32%
YS3334-G16	51.61%	59.65%	51.61%	58.06%	61.02%	58.33%	60.66%	56.45%	63.93%	56.45%	57.78%	3.95%	51.61%~63.93%	58.20%
YS3334-G22	75.00%	77.78%	76.47%	72.22%	54.84%	59.02%	61.02%	66.67%	72.73%	80.77%	69.65%	8.80%	54.84%~80.77%	72.48%
YS3334-F60	75.86%	66.67%	71.43%	73.77%	61.90%	60.32%	55.56%	53.13%	54.10%	56.67%	62.94%	8.48%	53.13%~75.86%	61.11%
YS3334-F25	62.07%	63.79%	56.45%	59.68%	66.67%	63.93%	70.00%	70.00%	63.33%	58.33%	63.43%	4.57%	56.45%~70.00%	63.56%
YS3334-F42	66.10%	59.32%	42.62%	79.66%	55.93%	56.67%	68.97%	66.67%	55.17%	66.10%	61.72%	10.05%	42.62%~79.66%	62.71%

Trichoderma area（S2-S1 cm ）	Sample 1	Sample 2	Sample 3	Sample 4	Sample 5
YS3334-CK	0.25	0.65	0.31	0.55	0.35
YS3334-G13	0.21	0.65	0.29	0.16	0.38
YS3334-G16	0.00	0.00	0.01	0.15	0.34
YS3334-G22	0.01	0.01	0.01	0.22	0.02
YS3334-F60	0.00	0.02	0.35	0.04	0.00
YS3334-F25	0.11	0.26	0.36	0.07	0.14
YS3334-F42	0.00	0.02	0.35	0.04	0.00

Note: DA = D2A - D1A; DB = D2B - D1B; RA = (DA / D1A)%; RB = (DB / D1B)%

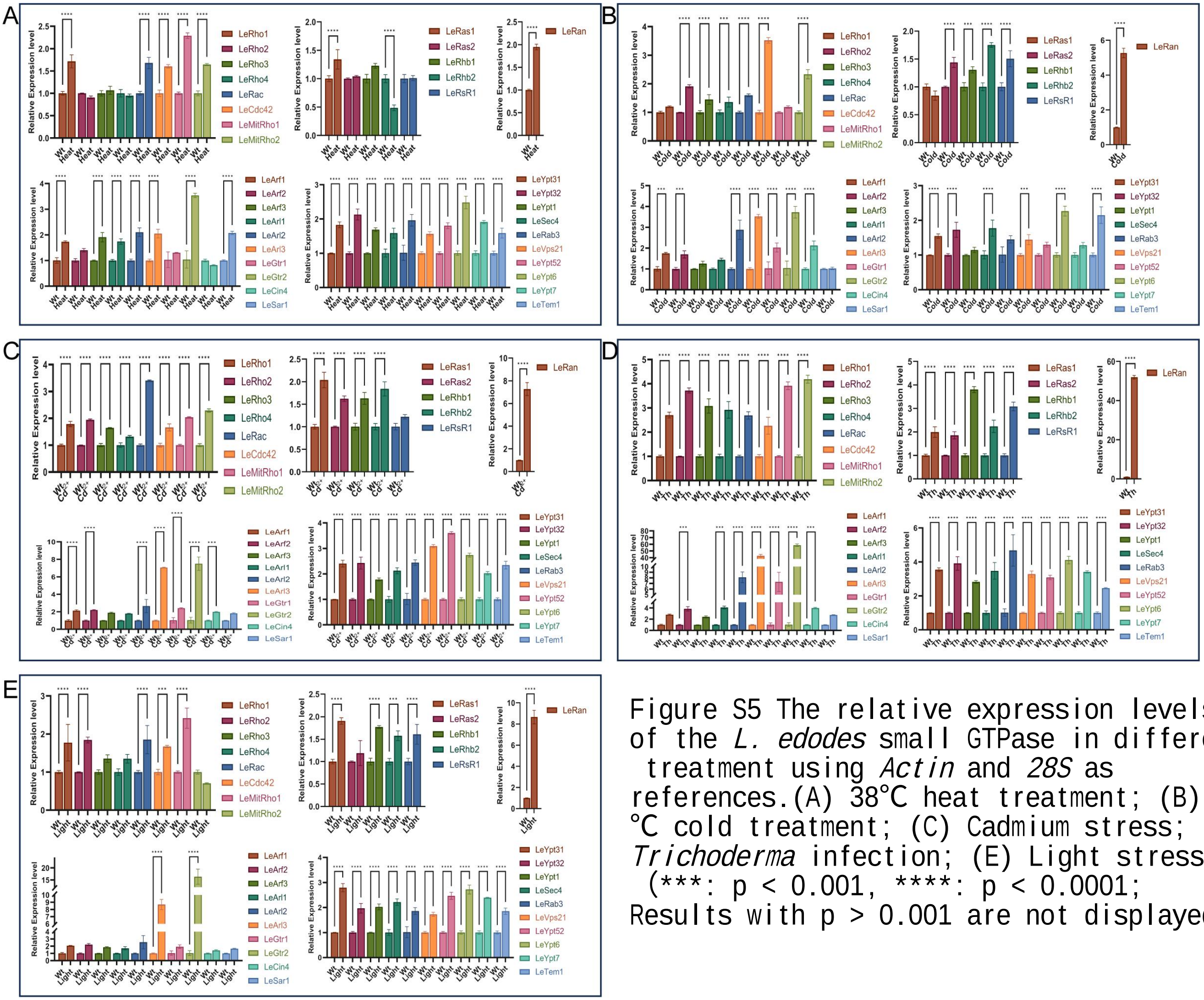

Figure S5 The relative expression levels of the *L. edodes* small GTPase in different treatment using *Actin* and *28S* as references.(A) 38℃ heat treatment; (B) 4 ℃ cold treatment; (C) Cadmium stress; (D) *Trichoderma* infection; (E) Light stress. (***: $p < 0.001$, ****: $p < 0.0001$; Results with $p > 0.001$ are not displayed)
